# The Toll-Dorsal Pathway Is Required for Resistance to Viral Oral Infection in *Drosophila*

**DOI:** 10.1371/journal.ppat.1004507

**Published:** 2014-12-04

**Authors:** Álvaro Gil Ferreira, Huw Naylor, Sara Santana Esteves, Inês Silva Pais, Nelson Eduardo Martins, Luis Teixeira

**Affiliations:** 1 Instituto Gulbenkian de Ciência, Oeiras, Portugal; 2 Department of Genetics, University of Cambridge, Cambridge, United Kingdom; Stanford University, United States of America

## Abstract

Pathogen entry route can have a strong impact on the result of microbial infections in different hosts, including insects. *Drosophila melanogaster* has been a successful model system to study the immune response to systemic viral infection. Here we investigate the role of the Toll pathway in resistance to oral viral infection in *D. melanogaster*. We show that several Toll pathway components, including Spätzle, Toll, Pelle and the NF-kB-like transcription factor Dorsal, are required to resist oral infection with *Drosophila* C virus. Furthermore, in the fat body Dorsal is translocated from the cytoplasm to the nucleus and a Toll pathway target gene reporter is upregulated in response to *Drosophila* C Virus infection. This pathway also mediates resistance to several other RNA viruses (Cricket paralysis virus, Flock House virus, and Nora virus). Compared with control, viral titres are highly increased in Toll pathway mutants. The role of the Toll pathway in resistance to viruses in *D. melanogaster* is restricted to oral infection since we do not observe a phenotype associated with systemic infection. We also show that *Wolbachia* and other *Drosophila*-associated microbiota do not interact with the Toll pathway-mediated resistance to oral infection. We therefore identify the Toll pathway as a new general inducible pathway that mediates strong resistance to viruses with a route-specific role. These results contribute to a better understanding of viral oral infection resistance in insects, which is particularly relevant in the context of transmission of arboviruses by insect vectors.

## Author Summary

Pathogenic microbes can enter their hosts through different routes. This can have a strong impact on which host defensive mechanisms are elicited and in disease outcome. We used the model organism *Drosophila melanogaster* to understand how resistance to viruses differs between infection by direct virus entry into the body cavity and infection through feeding on food with the virus. We show that the Toll pathway is required to resist oral infection with different RNA viruses. On the other hand this pathway does not influence the outcome of viral infection performed by injection. Together our results show that the Toll pathway has a route-specific general antiviral effect. Our work expands the role of this classical innate immunity pathway and contributes to a better understanding of viral oral infection resistance in insects. This is particularly relevant because insect vectors of emerging human viral diseases, like dengue, are infected through feeding on contaminated hosts.

## Introduction

Pathogens can infect their hosts through many different routes. In humans, for instance, microbes can directly enter the host through skin lesions or mediated by insect vectors. However, most of human infections start at mucosal surfaces of the respiratory, digestive or genital tracts. Pathogens specialize in different transmission strategies involving different host tissues. On the other hand, hosts mount distinct immune responses in different tissues, involving specialized cells and structures. Therefore, pathogen entry route can have a strong impact on the result of infection in animals, from humans to insects [1]–[4].

In *Drosophila melanogaster* oral or systemic infection with bacteria trigger different responses and have different outcomes (see [5] for review). Injection of bacteria into the haemocoel induces a systemic immune response based on the secretion of proteins into the haemolymph by the fat body [6]–[9]. Oral infections prompt a local immune response in the gut, and in some cases also a systemic response [10]–[13]. In both these responses the immune deficiency (Imd) signaling pathway can be activated and many antimicrobial peptides are produced [13]. However, these responses differ in other activated pathways and induced genes [13], [14]. Notably, the Toll pathway, a major mediator of systemic immune responses, is not involved in the gut local response. Injection of bacteria is generally more pathogenic than oral infection, with lower titres of bacteria being required for a lethal effect [2], [15]. Interestingly, the bacteria *Serratia marcescens* administered through oral infection can cross the gut barrier and enter the haemolymph, however these systemic bacteria have a lower pathogenicity than corresponding titres directly injected [15]. These findings indicate that natural infections lead to more structured and effective immune responses. These functional differences are also reflected in evolutionary processes since *Drosophila* adaptation to pathogenic bacteria is dependent on infection route [2].

Viral infections in insects have also been show to differ with infection route. For example, honeybees infected by Deformed Wing virus (DWV) through vertical transmission or horizontal oral transmission have no apparent disease symptoms. However, if horizontally transmitted by the parasitic *Varroa* mite, presumably from the mite saliva to the bee haemocoel, DWV is highly pathogenic [16]–[18]. Understanding the common and unique characteristics of insect defence against viral pathogens delivered through different routes is important in order to explain this differential pathogenicity. Moreover, resistance to viral oral infection in insects is also of particular interest since vectors of arboviruses are mainly infected through feeding on contaminated hosts.

*Drosophila melanogaster* has become an important model organism to study innate antiviral immunity in insects [19]–[21]. Some *Drosophila* viruses are vertically transmitted (e.g. Sigma virus) [22] and others can be infective by feeding, such as Nora virus [23], [24] and *Drosophila* C virus (DCV) [25]–[29]. ERK has recently been shown to be involved in resistance to RNA viruses by oral infection [29]. However, most of *D. melanogaster* antiviral immunity research has been done on systemic infection with viruses. The best characterized antiviral mechanism in *Drosophila* is the RNA interference (RNAi) pathway that has a strong influence on infection by a wide range of viruses, including RNA and DNA viruses [30]–[35]. Consistent with the important role of RNAi, several viruses express suppressors of this mechanism [30], [32], [36]. Other important mediators of antiviral protection are the intracellular bacteria *Wolbachia* [37], [38]. Presence of these endosymbionts increases resistance to several RNA viruses [37]–[40].

The role of classical *Drosophila* inducible immune pathways in antiviral defence seems less broad or well defined. The JAK/STAT pathway is required for resistance to DCV and Cricket Paralysis virus (CrPV) but not to other viruses [35], [41]. Similarly, mutants in the Imd pathway are less resistant to Sindbis virus and CrPV [42], [43] but not to DCV [44]. The role of the Toll pathway in antiviral immunity is less clear. This pathway is initiated by the binding of the cytokine Spätzle to Toll which triggers an intracellular signalling cascade involving the adaptor proteins dMyD88 and Tube and the kinase Pelle, and leads to activation of the NF-κB transcription factors Dorsal and Dorsal-related immunity factor (Dif) [45]–[47]. These transcription factors are normally sequestered in the cytoplasm and translocate to the nucleus upon Toll pathway activation. No phenotype was observed with DCV or Sindbis in *Dif* mutants or *Dif* and *dorsal* double mutants, respectively [43], [44]. However, *Dif* mutants are more susceptible to *Drosophila* X virus (DXV) [48]. On the other hand, the role of the whole pathway in resistance to DXV is not clear since loss-of-function mutants in *Toll* (*Tl*), *spätzle* (*spz*), *tube* (*tub*) and *pelle* (*pll*) show no phenotype [48]. Moreover, constitutive activation of the pathway, in a Toll gain-of-function mutant, also leads to higher susceptibility to DXV [48].

Data in other insects support an antiviral role for the Toll pathway. In honeybees *dorsal-1A* knockdown increases titres of DWV [49]. Also, in the mosquito *Aedes aegypti* the Toll pathway is induced upon ingestion of a dengue virus infected blood meal and inactivation of the pathway resulted in increased viral loads [50]. These studies raise the possibility that the Toll pathway is generally involved in the response to viruses in insects and prompt further analysis of its function in *Drosophila* antiviral immunity.

Here we investigate the role of the Toll pathway in immune response to several RNA viruses on *Drosophila melanogaster* comparing a natural infection route (i.e. by feeding) and systemic infection. We show that several Toll pathway components, including the extracellular cytokine Spätzle, the membrane receptor Toll, the kinase Pelle and the NF-kB-like transcription factor Dorsal, are required to resist natural viral infections in *Drosophila* but not systemic infection. These data provide evidence that the inducible Toll pathway has a route-specific general antiviral effect.

## Results

### Characterization of DCV oral infection in *Drosophila*

**Figure 1:**
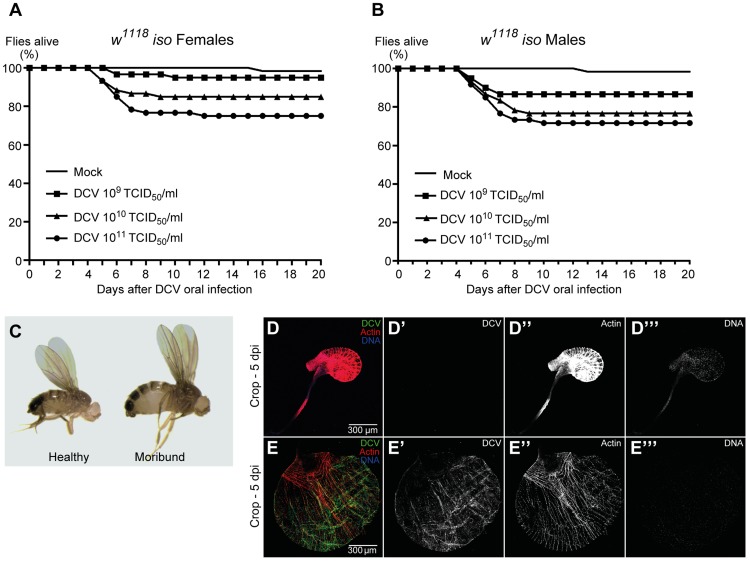
DCV oral infection can cause lethal infections. (A and B) Survival of adult flies after DCV oral infection. Sixty *w^1118^ iso* females (A) or males (B), 3–6 days-old flies were orally infected with DCV (10^9^, 10^10^, or 10^11^ TCID_50_/ml) or buffer (Mock), and the survival was monitored daily. Three independent experiments were performed, with similar results. Survival data of both genders and the three experiments were analysed together using the Cox proportional hazard mixed effect model and observed that dose is a highly significant factor (*p*<0.0001). (C–E) Flies were infected with DCV at 10^11^ TCID_50_/ml. (C) After DCV oral infection moribund flies become inflated while challenged but healthy-looking flies do not. (D–E) Moribund flies (E) exhibit an oversized and overinflated crop when compared with challenged healthy-looking flies (D). In moribund flies crop muscle cells are infected with DCV (E). Adult male crops were immunostained with antibody against DCV (green), actin marked with phalloidin (red) and DNA marked with TOTO3 (blue).

DCV is a non-enveloped virus with a single-stranded, positive-sense RNA genome that belongs to the *Dicistroviridae* family [51]. This virus is a natural pathogen of *D. melanogaster* that can be found in both wild and laboratory fly populations [22]. On most *Drosophila* studies using DCV the virus is injected directly into the body cavity, bypassing putative natural barriers and immune defences. In order to infect *Drosophila* flies with DCV through a natural route, we developed a protocol for oral DCV infection in adults. The protocol consisted in keeping adult flies with a mix of DCV and yeast for 24 hours in a vial. After this period, defined as 0 days post-infection (dpi), flies were transferred to vials containing standard *Drosophila* food and their survival scored daily. We found that DCV oral infection in adult DrosDel *w^1118^* isogenic (hereafter called *w^1118^ iso*) [52] flies can cause a lethal infection in both females and males, killing up to 25% of flies in 20 days (Fig. 1A, 1B and Dataset S1). We observed that flies started to die 5 to 6 dpi, similarly to infection by injection or pricking. We fitted the survival data with a Cox proportional hazard mixed effect model and compared the relative risk of dying of infected flies with non-infected controls (mock). In order to compare the different doses with each other we performed a Tukey's test on the resulting Cox hazard ratios. Lethality is dose-dependent since we observed that higher DCV doses induce significantly different higher lethality rates (Fig. 1A, 1B, S1 and Dataset S1).

We observed that both females and males that become lethargic and inflated die within one day (Fig. 1C). In order to identify the reason of the observed overinflated body, particularly the abdomen, we dissected these flies at 5 dpi. Moribund flies exhibit an oversized crop when compared with healthy flies (Fig. 1D, 1E and S2). Using immunofluorescence we detected DCV infecting crop-associated muscle cells (Fig. 1E), suggesting that viral infection of this visceral muscle is the reason of the crop oversize.

**Figure 2:**
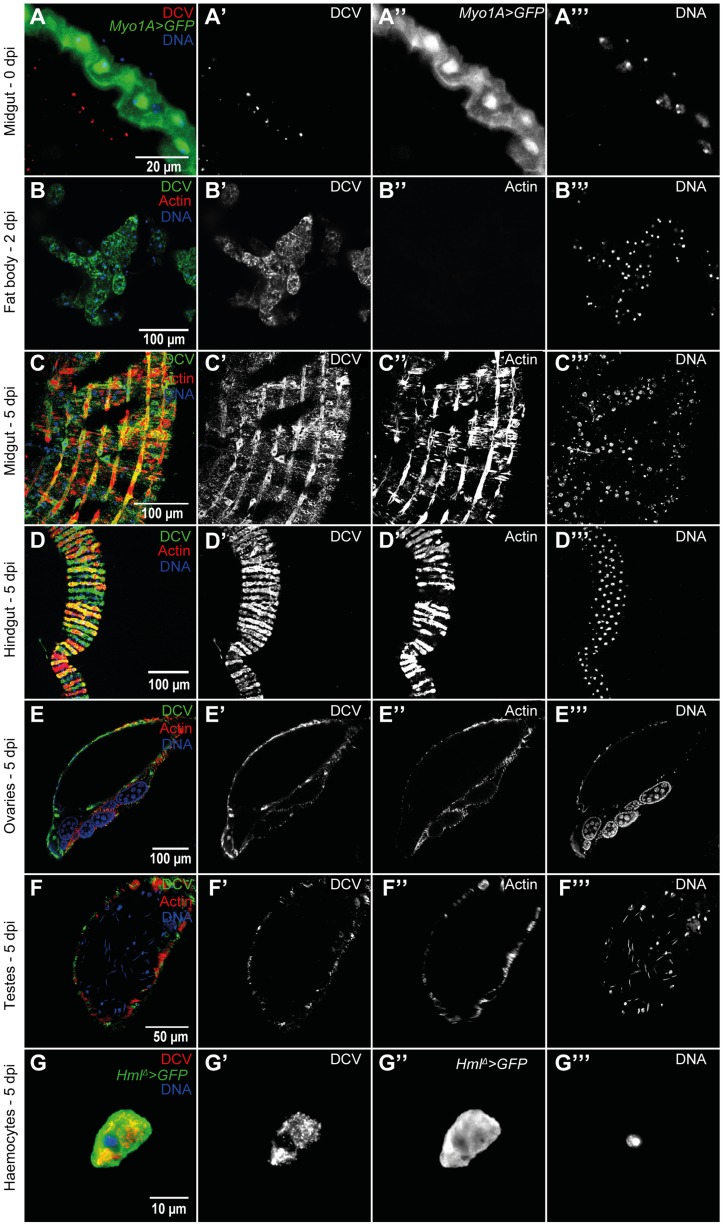
DCV tissue tropism upon oral infection. (A) DCV is present in midgut lumen at 0 dpi. Adult male guts were immunostained with antibody against DCV (red), epithelial enterocytes were marked with GFP expression (green) driven by *Myo1A-Gal4* and DNA marked with TOTO3 (blue). (B) Fat body is infected with DCV 2 dpi. Midgut (C) and hindgut (D) muscle cells are infected with DCV at 5 dpi. Muscle cells of the ovarian (E) and testis (F) peritoneal sheath are also infected with DCV 5 dpi. (B–F) DCV was immunostained with an antibody (green), actin marked with phalloidin (red) and DNA marked with TOTO3 (blue). Haemocytes (G) are infected with DCV 5 dpi. Haemocytes were marked with GFP expression (green) driven *by hml(delta)-Gal4*, DCV was immunostained with an antibody (red), and DNA marked DAPI (blue). All tissues were dissected from adult flies. DCV infections (10^11^ TCID_50_/ml) were performed in 3–6 days old flies.

To further characterize the course and the tropism of DCV upon oral infection we investigated which tissues were infected at 0 dpi (immediately after the 24 hours DCV exposure), 2 dpi and 5 dpi. We analysed oesophagus, crop, proventriculus, midgut, Malpighian tubules, hindgut, male and female reproductive organs, haemocytes, fat body, trachea and thorax skeletal muscle. At 0 dpi we were able to detect virus particles in the lumen of the midgut (Fig. 2A), indicating that the virus is reaching at least as far as the midgut. However, we were not able to detect any DCV infected cell, including epithelial and visceral muscle cells of the midgut (Fig. 2A). At 2 dpi the only tissue in which we could detect infection was the fat body (Fig. 2B). This DCV infection was confined to some regions of the fat body, mostly in the abdominal region. At 5 dpi DCV was also detected in the fat body (Fig. S3A) and the extent of the infection there was much greater than at 2 dpi. Analysis of flies 5 dpi also revealed the presence of DCV in the visceral muscle surrounding the crop, midgut and hindgut (Fig. 1E, 2C and 2D). Despite the presence of DCV in the midgut visceral muscle, we were not able to detect any virus in the gut epithelium. We also detected DCV in the muscle surrounding the Malpighian tubules at the junction point with the gut, but we were not able to detect DCV in the Malpighian tubules cells (Fig. S3B). The visceral muscle cells of the ovarian and testis peritoneal sheaths were also infected with DCV (Fig. 2E and F). We detected DCV in the abdominal muscle rarely (less than 1/40 flies) (Fig. S3C) but we never found DCV in thorax skeletal muscle (Fig. S3D). DCV was also detected in small sections of the tracheal system, mostly frequently in the abdominal region (Fig. S3E). Additionally, we observed that DCV was present in some circulating haemocytes (Fig. 2G). This could indicate that DCV efficiently infects haemocyte cells. Another possible explanation is that haemocytes phagocytose infected cells. Overall, these immunofluorescence results show that by oral infection, DCV infects specific tissues of *D. melanogaster*.

**Figure 3:**
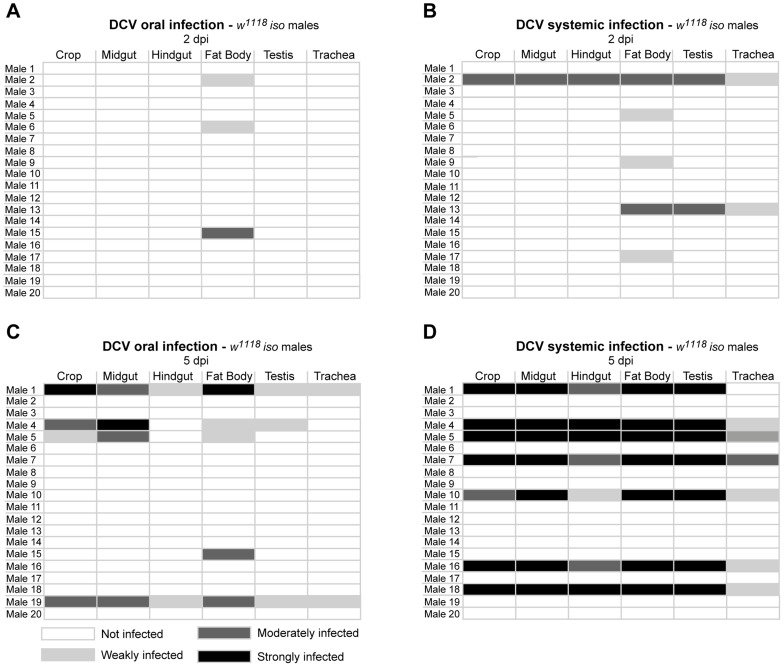
DCV oral and systemic infections have similar tissue tropism. (A and B) DCV tissue tropism 2 days after oral (A) and systemic (B) infection. (C and D) DCV tissue tropism 5 days after oral (C) and systemic (D) infection. DCV was at 10^11^ TCID_50_/ml for oral infection, 10^5^ TCID_50_/ml for systemic infection. Tissues of twenty adult males per condition were dissected and immunostained with an antibody against DCV, actin marked with phalloidin and DNA marked TOTO3. Oesophagus, crop, proventriculus, midgut, Malpighian tubules, hindgut, testes, fat body, trachea and thorax skeletal muscle tissues of every individual was analysed for DCV presence and the intensity of the infection by confocal microscopy. “Not infected” - DCV not detected in any part of the tissue observed. “Weakly infected” - DCV was detected in less than one third of the tissue. “Moderately infected” - DCV was detected in one to two thirds of the tissue. “Strongly infected” - DCV was detected in more than two thirds of the tissue. DCV infections were performed in 3–6 days old flies.

In order to compare DCV tropism upon oral and systemic infections we examined all the above tissues in 20 males 2 and 5 dpi for both infection protocols using immunofluorescence. For this we pricked flies with a relatively low dose of DCV (10^5^ TCID_50_/ml). Analysis of orally infected flies confirmed that at 2 dpi only the fat body was infected with DCV (Fig. 3A and Table 1). There was a restriction to the fat body in some systemically infected flies at 2 dpi, however in other flies the infection was also present in other tissues (Fig. 3B and Table 1). At 5 dpi DCV was detected in the same tissues for both virus-delivery methods (Fig. 3C, 3D and Table 1). These results show that independently of delivery route DCV tropism is largely the same, although it is less restricted to the fat body in the early stages of systemic infection.

**Table 1:** Presence of DCV in haemocytes of infected flies.

	*w^1118^ iso*		*pll^−/−^*	
	2 dpi	5 dpi	2 dpi	5 dpi
oral infection	0/10	2/10	4/10	5/10
systemic infection	1/10	3/10	3/10	8/10

3–6 days-old *w^1118^ iso* and *pll^−/−^* males were orally or systemically infected with DCV and analyzed 2 or 5 dpi for the presence of virus in haemocytes. 10 males were analyzed for each condition.

### Toll pathway mutant flies are less resistant to DCV oral infection

**Figure 4:**
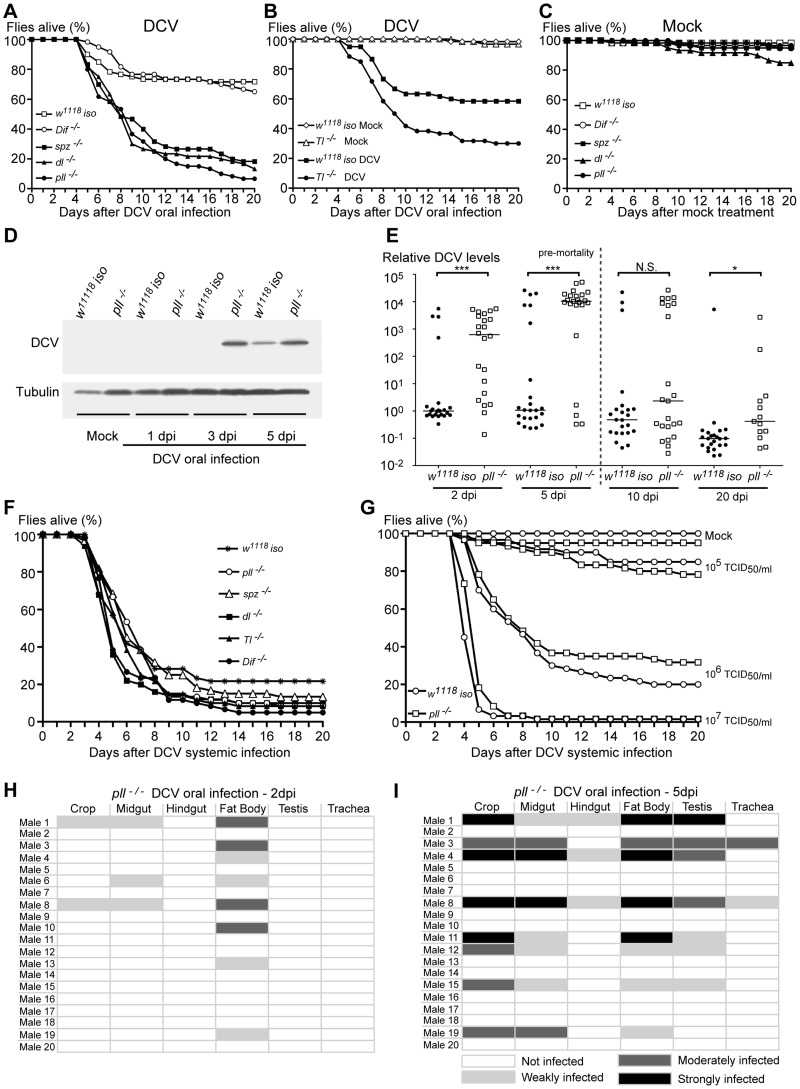
Toll Pathway mutant flies are less resistant to DCV oral Infection. (A and B) Survival of Toll pathway mutants upon DCV oral infection (10^11^ TCID_50_/ml) or buffer. Male flies *spz^−/−^* (*spz^4^*/*spz^4^*), *pll^−/−^* (*pll^2^*/*pll^21^*), *dl^−/−^* (*dl^1^*/*dl^1^*), D*if^−/−^* (*Dif^1^*/*Dif^1^*) (A) and *Toll^−/−^* (*Tl^rv1^*/*Tl^r3^*) (B) were compared to *w^1118^ iso*. *spz^−/−^*, *pll^−/−^ dl^−/−^* and *Tl^−/−^* flies were significantly different from *w^1118^ iso* (Cox proportional hazard mixed effect model, *p*<0.001 for all four lines). D*if^−/−^* mutant flies were not significantly different from *w^1118^ iso* (*p* = 0.331). (C) Survival of Toll pathway mutants upon mock treatment. None of the mutant lines were significantly different from *w^1118^ iso* (Cox proportional hazard mixed effect model, *p*>0.67), except *dl* (*p* = 0.003). (D) DCV protein levels after oral infection. 3–6 days old males of *pll^−/−^* and *w^1118^ iso* lines were orally infected with DCV (10^11^ TCID_50_/ml), collected 1, 3 and 5 days later for protein extraction, and probed in a Western blot with anti-DCV antibody (10 flies per sample). Anti-tubulin antibody was used as a loading control. (E) DCV RNA levels upon oral infection. 3–6 day old males of *pll^−/−^* and *w^1118^ iso* lines were orally infected with DCV (10^11^ TCID_50_/ml) and collected 2, 5, 10 and 20 days later for RNA extraction and RT-qPCR. 10 and 20 dpi infection samples are biased since they were collected after the major peak of DCV-induced mortality and therefore most highly infected flies have presumably died. Relative amount of DCV was calculated using host *Rpl32* mRNA as a reference and values are relative to median of *w^1118^ iso* samples at 2 dpi. Each point represents a sample (one male), and lines are medians of the samples. DCV loads are significantly different between *pll^−/−^* and *w^1118^ iso* line at 2, 5 and 20 dpi (Wilcoxon test, *p*<0.001, *p*<0.005, *p* = 0.25 and *p*<0.05 for 2, 5, 10 and 20 dpi, respectively). (F) Survival of Toll pathway mutants upon DCV systemic infection (pricked at 10^7^ TCID_50_/ml). None of the mutant lines were significantly different from *w^1118^ iso* (Cox proportional hazard mixed effect model, *p*>0.1). (G) Survival of *pll^−/−^* and *w^1118^ iso* male flies to different doses of DCV systemic infection. (10^5^, 10^6^ and 10^7^ TCID_50_/ml). *pll^−/−^* flies were not significantly different from *w^1118^ iso* (Cox proportional hazard mixed effect model, *p* = 0.840, *p* = 0.626 and *p* = 0.085, respectively). (H and I) DCV tissue tropism of *pll^−/−^* flies upon oral infection, 2 dpi (H) and 5 dpi (I). Twenty adult males per condition were dissected and immunostained with an antibody against DCV and analysed as before. (A, B, C, F, G) For all survival experiments, sixty 3–6 days old males, per line or condition, were infected with DCV or buffer, and their survival was monitored daily. Survival assays for oral infections were performed thrice for *pll*, *spz*, an *dl* mutants, and twice for *Dif* and *Tl* mutants. Survival assays of systemic infection in panel F were performed twice. Survival data of all replicates were analysed together using Cox proportional hazard mixed effect models.

In order to analyse the role of the Toll pathway in the response to viral oral infection we tested a collection of mutants in different genes of the Toll pathway: *spz* (*spz^4^*/*spz^4^*), *Tl* (*Tl^rv1^*/*Tl^r3^*), *pll* (*pll^2^*/*pll^21^*), *dorsal* (*dl^1^*/*dl^1^*) and *Dif* (*Dif^1^*/*Dif^1^*). To limit putative effects of different genetic backgrounds each mutation was introgressed into the *w^1118^ iso* background. This introgression was done by chromosome replacement and backcrossing (see Materials and Methods). We orally infected these mutant lines with DCV and their survival was compared to the control line *w^1118^ iso*. Flies mutant in the genes *spz*, *Tl* and *pll* were more susceptible to DCV oral infection than *w^1118^ iso* control flies (Fig. 4A, 4B, S4A, S4B and Datasets S2, S3, S4). For *pll* mutants we further show increased sensitivity, compared with *w^1118^ iso*, at several DCV infection doses and dose-dependent lethality (Fig. S5A–D and Dataset S5). Mutations in the genes encoding the two NF-**κ**B homologues known to be downstream of the Toll pathway give different results. *Dif* mutants do not show a phenotype in this assay and are as sensitive to DCV infection as the *w^1118^ iso* control flies while *dl* mutants show high susceptibility, to the same degree as *spz* and *pll* mutants (Fig. 4A and S4A). These results contrast with the requirement of *Dif* but not *dl* in adult flies to resist bacteria and fungi [46], [53]–[56]. The high lethality observed for the several mutations in genes of the Toll pathway when compared to the control background are a consequence of DCV infection, since in the absence of viral infection and in the timeframe of this analysis these mutations have no effect on survival, except for the *dl* mutant that seems to be slightly deleterious by itself (Fig. 4C and S4C). In summary, these data show that the Toll pathway is important to survive DCV infection and Dorsal, but not Dif, is the downstream transcription factor required.

To investigate whether the increased lethality rates of *pll*-deficient flies was due to decreased resistance or tolerance to DCV we analysed the viral levels by Western blot at 1, 3 and 5 dpi. We observed that at 3 and 5 dpi *pll* mutant flies had more viral protein than *w^1118^ iso* flies (Fig. 4D and S6). We confirmed these results by measuring DCV RNA levels of single flies at 2, 5, 10 and 20 dpi using reverse transcription quantitative PCR (RT-qPCR). A greater number of *pll* mutant flies exhibited high quantities of DCV RNA when compared with *w^1118^ iso* flies at 2 and 5 dpi (Fig. 4E, S5E and Dataset S6). DCV titres are significantly different between these lines at these days and the median of viral RNA load was approximately one thousand to ten thousand times higher in *pll* mutant flies (Fig. 4E and S5E). All the flies analysed are infected with DCV, even the ones that survive the infection for 20 days. This shows that there is no clearance of the virus in this timeframe. These results show that a mutant in the Toll pathway has lower resistance to DCV upon oral infection.

In order to investigate if the Toll pathway is also required to resist DCV systemic infection we pricked Toll pathway mutants and *w^1118^ iso* flies with DCV and followed their survival for 20 days. We found that Toll pathway mutant lines were not more susceptible to DCV systemic infection when compared with *w^1118^ iso* flies (Fig. 4F, S7A–C and Datasets S7, S8). We further analysed the *pll* mutant infected at several doses in order to rule out a dose-specific lack of effect. *pll* mutant was not more sensitive than *w^1118^ iso* to DCV systemic infection in any of the doses (Fig. 4G and Dataset S9). These results suggest that, contrary to oral infection, the Toll pathway is not important in the immune response to DCV systemic infection.

To explore whether Toll pathway mutant flies have altered patterns of infection, we analysed DCV tropism at 2 and 5 dpi in *pll* mutant flies after oral infection. As before, 20 males were analysed per time point. In this mutant we can detect DCV in a higher proportion of flies at 2 dpi and 5 dpi than *w^1118^ iso* (40% compared with 15 or 25%, Fig. 3A, 3C, 4H, 4I and Table 1), in agreement with the RT-qPCR data. In contrast to *w^1118^ iso* flies (Fig. 3A), at 2 dpi DCV is not restricted to the fat body and can also be detected in muscle surrounding the crop and the midgut (Fig. 4H). At 5 dpi *pll* mutant flies showed the same DCV tropism as the observed in *w^1118^ iso* (Fig. 3C and 4I). We were also unable to detect DCV in crop or midgut epithelial cells in *pll* mutants. These results show that although DCV seems to be spreading faster in *pll* mutant flies than in *w^1118^ iso*, overall there is no difference in DCV tropism.

### Lack of interaction between *Wolbachia* and other microbiota with Toll resistance to viruses

**Figure 5:**
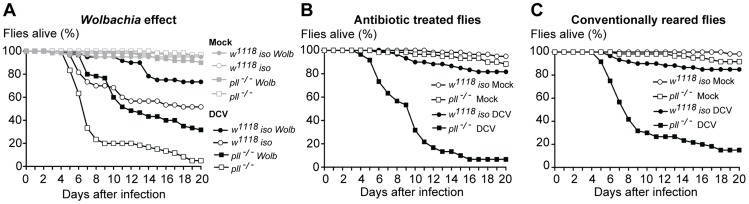
Lack of interaction between *Wolbachia* and other microbiota with Toll resistance to viruses. (A–C) Sixty 3–6 days old males of each line were orally infected with DCV (10^11^ TCID_50_/ml) or buffer (Mock), and survival was monitored daily. Survival data was fitted with a Cox proportional hazard mixed effect model. (A) *Wolbachia* protection to DCV oral infection does not require the Toll pathway. There is no interaction between *Wolbachia* and genotype (*p* = 0.67). (B–C) Survival of antibiotic treated (B) and conventionally reared (C) *pll^−/−^* and *w^1118^ iso* flies after DCV oral infection. There is no effect of antibiotic treatment in fly survival (*p* = 0.28). *pll^−/−^* flies show increased mortality relative to *w^1118^ iso* flies in both antibiotic treated or conventionally reared conditions (p<0.001 in both conditions).

*Wolbachia* induces resistance to infection by RNA viruses in *D. melanogaster* and other insects [37], [38], [57]. In mosquitoes this protection has been suggested to be dependent on the Toll pathway [58]. To test this hypothesis in *D. melanogaster* we compared the survival of *w^1118^ iso* and *pll*-deficient flies, infected and non-infected with *Wolbachia*. The results show that in *D. melanogaster Wolbachia* also protects against viral oral infection (Fig. 5A, S8A–C and Datasets S10, S11, Cox Proportional Hazards Model, *p*<0.001). We observed that *Wolbachia* protects *pll* mutants against DCV infection to the same extent as *w^1118^ iso* flies (Fig. 5A and S8A–C). The *pll* mutation does reduce survival of *Wolbachia*-positive flies when orally infected with DCV but to the same extent as in *Wolbachia*-free flies and there is no interaction between the two factors (Cox Proportional Hazards Model, Wolbachia*Genotype interaction; *p* = 0.67). The same lack of interaction is observed for systemic DCV infection (*p* = 0.69). Therefore in *D. melanogaster* the Toll pathway is not absolutely required for *Wolbachia* protection to DCV, confirming previous data with dengue virus [59].

Other *Drosophila*-associated microbiota could also indirectly affect DCV oral infection and Toll pathway mediated protection. The Toll pathway could, for instance, be important to control a secondary infection with bacteria upon viral infection induced damaged. *w^1118^ iso* and *pll*-deficient flies were raised and maintained with antibiotics and susceptibility to DCV was compared with conventionally-reared flies (Fig. 5B and C, S8D–F and Dataset S12). There is no significant effect of the antibiotic treatment on the susceptibility to viruses (Cox Proportional Hazards Model; *p* = 0.28) and *pll*-deficient flies are still more susceptible to DCV infection in the absence of bacteria (Cox Proportional Hazards Model; *p*<0.001). Hence, in our experimental setup the *Drosophila*-associated microbiota does not play a role in the susceptibility to DCV oral infection or Toll-mediated resistance.

### Toll pathway mutant flies are less resistant to oral infection with several viruses

**Figure 6:**
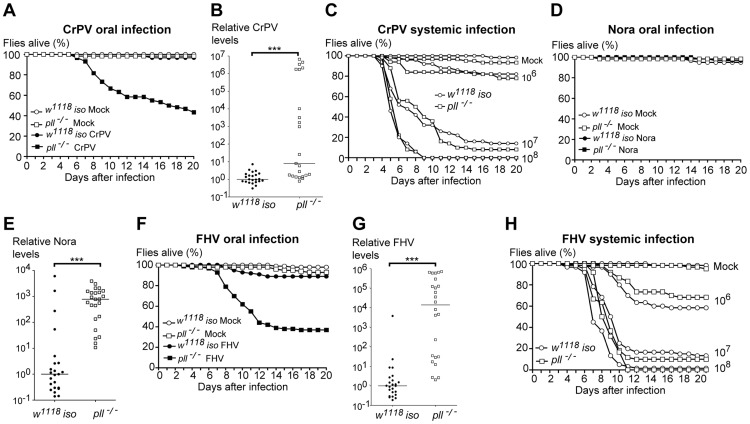
Toll Pathway mutant flies are less resistant to other RNA viruses oral infection. (A) Survival of *pll^−/−^* and *w^1118^ iso* flies after CrPV oral infection (1.76×10^10^ TCID_50_/ml) or buffer. *pll^−/−^* flies were significantly more sensitive to CrPV than *w^1118^ iso* (Cox proportional hazard mixed effect model, *p*<0.001). (B) CrPV RNA levels in *pll^−/−^* and *w^1118^ iso* flies upon oral infection (1.76×10^10^ TCID_50_/ml). CrPV loads are significantly different between *pll^−/−^* and *w^1118^ iso* line (Wilcoxon test, *p*<0.005). (C) *pll^−/−^* and *w^1118^ iso* flies were systemically infected with CrPV at three different concentrations (10^6^, 10^7^, 10^8^ TCID_50_/ml). *pll^−/−^* mutant flies were not more susceptible to CrPV systemic infection than *w^1118^ iso* control flies (Cox Proportional Hazards Model, *p* = 0.966, *p* = 1.000 and *p* = 0.974, respectively). (D) Survival of *pll^−/−^* and *w^1118^ iso* flies upon Nora oral infection or buffer. *pll^−/−^* flies were not more sensitive than *w^1118^ iso* (Cox proportional hazard mixed effect model, *p* = 0.887). (E) Nora RNA levels upon oral infection. Nora loads are significantly different between *pll^−/−^* and *w^1118^ iso* line (Wilcoxon test, *p*<0.005). (F) Survival of *pll^−/−^* and *w^1118^ iso* flies upon FHV oral infection (10^10^ TCID_50_/ml) or buffer. *pll^−/−^* flies were significantly more sensitive than *w^1118^ iso* (Cox proportional hazard mixed effect model, *p*<0.001). (G) FHV RNA levels upon oral infection (10^10^ TCID_50_/ml). FHV loads are significantly different between *pll^−/−^* and *w^1118^ iso* line (Wilcoxon test, *p*<0.005) (in the other independent replicate the difference in medians is 20-fold and *p* = 0.05). (H) *pll^−/−^* and *w^1118^ iso* flies were systemically infected with FHV at three different concentrations (10^6^, 10^7^, 10^8^ TCID_50_/ml). *pll^−/−^* mutant flies were not more susceptible to FHV systemic infection than *w^1118^ iso* control flies (Cox Proportional Hazards Model, *p* = 0.819, *p* = 0.709 and *p* = 0.225, respectively). For survival experiments (A, C, D, F and H) sixty 3–6 days old males of each line per treatment were used and survival was scored daily. Survival experiments for oral infections were performed thrice, yielding similar results. Survival data of all replicates was analysed together using the Cox proportional hazard mixed effect model. For viral loads experiments (B, E, G) 3–6 days old males of each line were orally infected with the virus of interest and collected 5–6 dpi for RNA extraction and RT-qPCR. Relative amount of virus was calculated using host *Rpl32* mRNA as a reference and values are relative to the median of the *w^1118^ iso* samples. Each point represents the relative virus amount of a single fly and lines are medians of these values. All viral loads experiments were performed twice yielding similar results.

To test the specificity of the Toll pathway in *D. melanogaster* antiviral immune response, we tested its requirement for resistance to other insect RNA viruses. Cricket Paralysis virus (CrPV) is closely related to DCV, also belongs to the *Dicistroviridae* family, and causes a lethal infection in adult flies [32], [42], [60]. Upon CrPV oral infection we observed that *pll*-deficient flies were more susceptible than control flies (Fig. 6A, S9A and Dataset S13). As with DCV oral infections, we found that a greater number of *pll* mutant flies exhibited higher amounts of CrPV RNA when compared with *w^1118^ iso* flies (Fig. 6B and Dataset S14). *pll*-deficient flies showed the same susceptibility to CrPV systemic infection as control flies at different viral infection titres (Fig. 6C, S9B and Dataset S15).

We also tested whether *pll*-deficient flies are more sensitive to Nora virus oral infections. Nora virus is a picorna-like, non-enveloped virus, with a positive-sense single-stranded RNA genome [23]. This virus naturally infects *D. melanogaster* and causes persistent infection without any evidence of pathology [23], [24]. Nevertheless, we compared the lethality rates of *pll*-deficient flies with *w^1118^ iso* control flies upon Nora virus oral infection. As show in Fig. 6D and S9C (Dataset S16), we did not observe any lethality associated with Nora oral infection, even in *pll* mutants. However, when we measured Nora RNA levels of single flies 5 dpi we found that a greater number of *pll* mutant flies exhibited high amounts of viral RNA when compared with *w^1118^ iso* flies (Fig. 6E and Dataset S14).

Finally, we investigated the importance of Toll pathway in the immune response to Flock House virus (FHV). FHV is a non-enveloped, positive-sense RNA virus that belongs to the *Nodaviridae* family of insect virus [61]. Although FHV is not a natural pathogen of *D. melanogaster* it can replicate and cause lethality in adults when injected [31]. *pll*-deficient flies were more susceptible to FHV oral infection than *w^1118^ iso* control flies (Fig. 6F, S9D and Dataset S17) and had higher levels of viral RNA (Fig. 6G and Dataset S14). We also tested whether *pll* mutant flies were more susceptible to FHV systemic infection. In concordance with the DCV results, *pll* mutant flies were not more susceptible to FHV systemic infection when compared with *w^1118^ iso* flies across several doses of infection (Fig. 6H, S9E and Dataset S18).

These analyses with different viruses indicate that the Toll pathway is required to resist a broad range of RNA viruses. Moreover, this requirement seems to be specific to oral infection and not relevant in the context of a systemic infection. To establish whether the increased sensitivity to viral infection extended to other pathogens, we orally infected *pll*-deficient flies with *Pseudomonas entomophila* and compared their survival to *w^1118^ iso*. *pll*-mutant flies were not more susceptible to these Gram-negative bacteria than *w^1118^ iso* (Fig. S10A and Dataset S19). This was expected since the Toll pathway is not required for the transcriptional immune response to Gram-negative bacteria gut infection [13]. We also analysed the feeding rates of *pll* mutant and *w^1118^ iso* flies. When exposed to DCV mixed with yeast both lines had the same feeding rate (Fig. S10B and Dataset S20). These data show that Toll pathway mutant flies are not generally more susceptible to oral infection by all pathogens. Testing further pathogens will allow assessing if increased susceptibility of Toll pathway mutants is restricted to viruses.

### DCV infection induces activation of the Toll pathway

**Figure 7:**
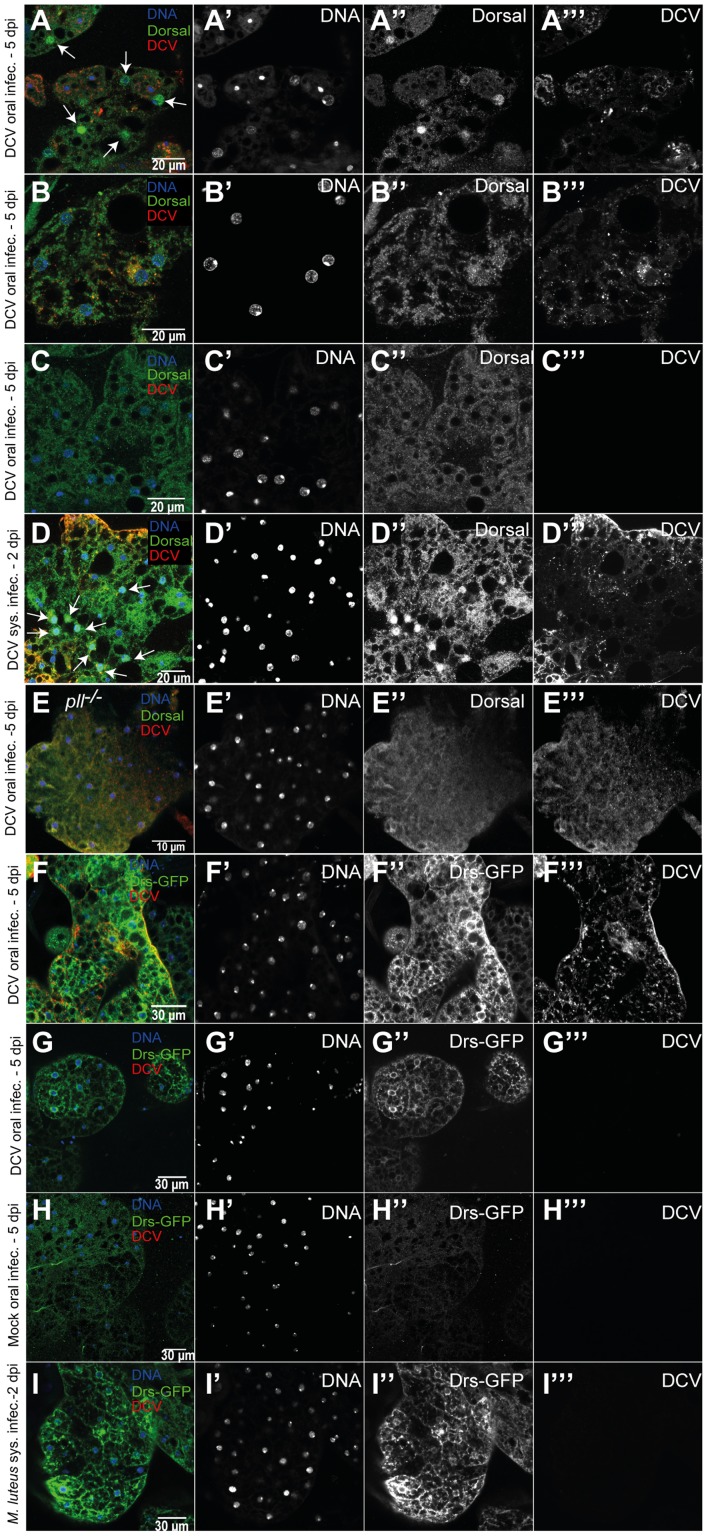
Toll pathway activation by DCV infection. (A–C) Dorsal localization in fat body cells 5 days after DCV oral infection (10^11^ TCID_50_/ml) of *w^1118^ iso* flies. All three fat body regions shown were dissected from the same fly and are representative of 14 DCV-positive flies (out of 20 total flies analysed). (A) DCV infected fat body region with nuclear import of Dorsal (white arrows). Nuclear import of Dorsal was observed in 4 out of the 14 DCV positive flies (B) DCV infected fat body region without nuclear import of Dorsal. (C) Fat body region not DCV infected without nuclear import of Dorsal. (D) Dorsal localization in fat body cells 2 days after DCV systemic infection (10^7^ TCID_50_/ml) of *w^1118^ iso* flies, showing nuclear import of Dorsal (white arrows). Nuclear import of Dorsal was observed in 5 of 10 DCV positive flies. (E) Dorsal localization in fat body cells of *pll^−/−^* flies after DCV oral infection (Dorsal nuclear import was seen in 0 out of 16 DCV positive flies) (A–E) Adult male fat body was immunostained with an antibody against Dorsal (green), an antibody against DCV (red), and DNA was marked with DAPI (blue). (F–G) *Drs*-GFP expression in fat body 5 days after DCV oral infection. Both fat body regions shown were dissected from the same fly. (H) *Drs*-GFP expression in fat body after 5 days mock oral infection. (I) *Drs*-GFP in fat body after 2 days *Micrococcus luteus* oral infection. (F–I) Adult male fat body regions were immunostained with antibody against DCV (red), antibody against GFP (green), and DNA marked with TOTO3 (blue). DCV infections were performed in 3–6 days old flies.

Since we observed that flies mutant in genes of the Toll-Dorsal pathway have increased sensitivity to DCV oral infection, we investigated whether Dorsal is activated during viral infection. We probed if Dorsal was translocated from the cytoplasm to the nucleus upon DCV infection by using an antibody specific against its C-terminal domain [62] (Fig. S11A and B). At 5 days after oral infection, but not 2 dpi, we were able to detect nuclear import of Dorsal in fat body cells infected with DCV (20 flies were analysed in each time point) (Fig. 7A). This is only observed in infected cells although many fat body cells infected with DCV do not show Dorsal nuclear localization (Fig. 7B). However, we never detected Dorsal enrichment in the nuclei of non-infected fat body cells, even in infected flies (Fig. 7C and S11A). This nuclear translocation upon DCV infection seems specific to the fat body since we do not observe it other tissues, including gut epithelial and muscle cells (in the same 2 dpi and 5 dpi samples) (Fig. S11C and S11D). We have also failed to detect Dorsal translocation in haemocytes of *w^1118^ iso* flies orally or systemically infected (the *w^1118^ iso* infected flies analysed in Table 1) (Fig. S11E). We also analysed Dorsal localization after DCV systemic infection (2 dpi and 5 dpi, 10 flies each). Dorsal is translocated to the nuclei of fat body cells in response to DCV infection at 2 dpi (Fig. 7D) but not in epithelial or muscle cells of the gut in the same flies. Finally, we tested Dorsal translocation upon oral viral infection in a *pll* mutant line (Fig. 7E). We do not see Dorsal translocation in 16 DCV infected flies that are *pll^−/−^* but we see translocation in 4 out of 14 infected *w^1118^ iso* control flies (chi-square test, p = 0.037). This shows that Dorsal translocation in response to viral infection is dependent on the Toll pathway. In summary, these results show that Dorsal is translocated from the cytoplasm to the nucleus in fat body cells in response to DCV infection, suggesting that the Toll pathway is involved in an antiviral inducible immune response.

*Drosomycin* (*Drs*) encodes an antimicrobial peptide and is a target gene of immune activation of the Toll pathway [7]. We probed expression of a *Drs* reporter gene [47] in response to viral infection. We observed *Drs*-GFP expression in the fat body of 8 out of 8 DCV infected flies but not in gut muscle or epithelium (Fig. 7F and G and S11F and G). Out of 8 non-infected flies none showed activation of *Drs-GFP* expression (Fig. 7H). The *Drs*-GFP fat body expression is present in infected and non-infected cells in DCV-infected flies, unlike Dorsal translocation, indicating a systemic activation of Toll pathway. This result further shows that the Toll pathway is activated in the fat body upon viral infection.

In order to test if inactivation of the Toll pathway in the fat body or other tissues (muscle, visceral muscle, enterocytes and haemocytes) would increase sensitivity to viruses we expressed three RNAi constructs for *pll* with different drivers and compared survival after DCV oral infection with control (Fig. S12 and Dataset S21). We failed to see any significant increase in lethality upon viral infection in these lines. Based on this negative result with RNAi it is not possible to conclude on the need of the Toll pathway anti-viral response in specific tissues.

## Discussion

In *Drosophila* the Toll pathway plays a fundamental role in the response to systemic infection by fungi and bacteria [7], [54], [63]. Here we show that this pathway is also required to resist oral viral infections. Mutants in genes that encode the ligand Spätzle, the Toll transmembrane protein receptor, the cytoplasmic kinase Pelle and the NF-**κ**B transcription factor Dorsal succumb faster to DCV infection and have higher titres of this virus. This demonstrates that a functional canonical Toll pathway is required for flies to survive a natural viral infection.

Two very similar NF-**κ**B homologues, Dif and Dorsal, can be downstream of the Toll pathway in flies. Our genetic analysis shows that Dorsal but not Dif is required for viral resistance. In contrast, Dif but not Dorsal is required for adult systemic response to infection by bacteria and fungi [7], [46], [53], [63] and has been regarded as the Toll pathway transcription factor involved in adult immune systemic response. Nonetheless, other data also indicate a role for Dorsal in the immune response. In larvae both Dif and Dorsal are translocated into fat body cell nuclei upon systemic infection with bacteria [6], [45], [64] and Dorsal is upregulated in infected larvae [65]. In larvae Dif and Dorsal may be redundant in resistance to bacteria with the double mutant being very susceptible to normal *Drosophila*-associated microbiota [66]. Dif and Dorsal can form homo and heterodimers and these recognize different DNA sequences and differentially activate target genes [67], [68]. However, overexpression of one or the other transcription factor many times is sufficient to rescue mutant or double mutant phenotypes [66], [69] as well as activate expression of antimicrobial peptides [69]. Overall, the exact role and contribution of either transcription factor in the several types of immune responses of *Drosophila* is not known. On the other hand Dorsal also has a clear role in development in early embryogenesis, which Dif does not seem to share [45], [70]. The Toll pathway, although not necessarily through Dorsal, also has a role in muscular and neuromuscular development [71]–[75] and hematopoiesis [76]. This raises the possibility that the phenotypes we observed are due to development problems. This hypothesis would be particularly relevant for oral infection with viruses if development problems were to lead to alterations in feeding. However, we observe no differences in feeding rates between *pll* mutants and control. Moreover, we show that *pll* mutants are not more susceptible to oral infection with a bacterial pathogen. These data indicate that it is not a major digestive system development problem that leads to lower resistance to oral viral infection. We cannot absolutely rule out a development problem; however, we detect Dorsal translocation into the nuclei of DCV infected fat body cells and expression of a *Drosomycin* reporter gene in the fat body of infected flies. This shows that the Toll-Dorsal pathway is induced upon viral infection and, together with the genetic data, strongly supports a Toll pathway mediated anti-viral response. Identification of the target genes of the Dorsal transcription factor after viral infection will be important in the future, as well as understanding how they contribute to resistance to viruses.

During embryonic development and systemic immune response to fungi and bacteria the extracellular pro-Spätzle is proteolytically cleaved, leading to binding to the Toll receptor and activation of the pathway. In the case of infection, specific pattern recognition receptors present in the haemolymph are activated by microbial ligands and start a proteolytic cascade that culminates in pro-Spätzle cleavage [55], [56], [77]. Fungal and bacterial proteases can also lead to Spätzle cleavage through a different proteolytic cascade involving Persephone [77], [78]. At this point it is unclear how activation of the Toll pathway by viral infection works and it probably differs significantly from activation by bacteria and fungi. Putative pathogen associated molecular patterns associated with viruses and recognized by *Drosophila* must be different from the cell wall components of bacteria and fungi involved in Toll pathway activation. Moreover, viruses are intracellular parasites while the previously studied microbial elicitors of the Toll pathway are extracellular and present in the haemolymph. Previous work has shown that in *Drosophila* the Toll pathway also responds to tumours [79] and to a block in apoptosis, via Persephone [80], while in mosquitoes it can be activated by reactive oxygen species [58]. Viral infection could be indirectly detected by the Toll pathway through recognition of tissue damage and share a mechanism of activation with the above situations. *Drs* expression in response to viral infection is widespread in the fat body of infected flies and not restricted to infected cells. This indicates that Spätzle activation is systemic upon viral infection and that the Toll pathway is generally activated in the fat body of these flies. This is in agreement with previous published data showing up-regulation of *Drs* and Toll pathway genes upon DCV infection [28], [35]. As a further layer of complexity, our results show that Dorsal translocation is restricted to viral infected cells and is not observed throughout the fat body. This is at odds with a systemic activation of Spätzle and how the Toll pathway responds to bacteria and fungi. It is possible that Dorsal is activated throughout the fat body but that is not visible in the translocation assay. However, Dorsal activation and translocation to the nucleus may depend on Toll activation and a second cell-autonomous signal. In mammals RIG-I-like receptors (RLRs) and NOD-like receptors are involved in cell-autonomous activation of innate immunity in response to viral infection. There are no homologues of these cytoplasmic pattern recognition receptors in *Drosophila*. However, Dcr2 has a helicase domain homologous to helicase domains in RLRs and has been suggested to act as a pattern recognition receptor in *Drosophila* [81]. Toll-like receptors in mammals are also able to detect viral infection through binding to nucleic acids in vesicular compartments. Toll-7 in *Drosophila* can bind vesicular stomatitis viruses and induce antiviral autophagy [82]. Unravelling the signal that leads to Dorsal translocation in virally infected cells will be important to understand antiviral immunity in *Drosophila*.

Our results show that the increased lethality rates observed in the Toll pathway deficient flies are associated with higher DCV loads. Thus, the Toll pathway is involved in resistance to viruses. Furthermore, we demonstrate in this study that Toll requirement to control viral loads is not specific to DCV and extends to other RNA viruses, such as FHV, CrPV and Nora virus. Previous work did not see an effect of a *pll* mutant in a Nora virus infection assay [83]. The difference in our results may be due to different control of the genetic background or differences in the assay. We analysed the response to a new Nora virus oral infection while Habayeb and colleagues analysed the capacity to clear the viruses in a chronically infected *Drosophila* stock [83]. The median increase in viral titres we observe in *pll* mutants can be up to ten thousand fold. The magnitude of the difference is comparable or higher to differences between wild type flies and RNAi mutants [30]–[33] and between flies with and without *Wolbachia* [37]. The strength and generality of the interaction between the Toll pathway and viruses indicates that this is a major antiviral pathway in *Drosophila*. This is consistent with previous studies showing Toll pathway antiviral effect in mosquitoes and honeybees [49], [50].

The increased sensitivity to viruses in Toll pathway mutants is only manifested upon oral infection and not systemic infection. This is not a result of different infection titres with the two modes of infection because Toll pathway mutants are not more sensitive to a low dose of virus by systemic infection. Therefore, we have identified a pathway with a route-specific role. Nonetheless, we observe Dorsal nuclear translocation in fat body cells after both routes of infection. This indicates that the pathway is activated regardless of type of infection but it is only effective in a scenario of oral infection. In order to understand the differential requirement of the Toll pathway we performed a detailed analysis of the dynamics of DCV oral and systemic infections. Overall we found no major differences in the tissue distribution of DCV between the two infection routes. In both DCV is present in the fat body, trachea and visceral muscle of the crop, midgut and hindgut, and gonads. Although we can detect DCV particles in the midgut lumen shortly after oral ingestion, we could not determine its point of entry. We were unable to detect DCV infection in the epithelium of the digestive system at any time point. This could indicate that the DCV is transported across gut epithelial cells to the body cavity (haemocoel) without infecting the epithelial cells themselves. Transcytosis of virions has been described in mammals and insects [84]–[86]. An alternative explanation would be that DCV rapidly kills infected epithelial cells, therefore hindering their detection. Apoptosis of midgut cells following viral infection has been observed in *Drosophila* and in mosquitoes [87], [88]. However, a recent study in *Drosophila* reported that upon oral ingestion DCV was able to infect midgut epithelial cells [29]. The difference between these results may reflect differences in the feeding protocol: Xu and colleagues continually exposed flies to DCV for several days [29], while we only infect flies for one day. In our setup the fat body seems to be the first tissue to be infected; all infected flies have DCV in the fat body and some infected flies only have DCV in the fat body. This is more evident in orally infected flies that at 2 dpi only have DCV in this tissue. This may reflect a difference in the dynamics of the two infection routes and in systemic infected flies DCV seems to disseminate faster. The detection of Dorsal translocation only in fat body cells and the probable early restriction of DCV to this tissue when delivered by oral infection may be part of the explanation of the differential requirement of the Toll pathway in the two routes of infection.

Our results show that the Toll pathway is required to resist viral infections, which adds to the previously known requirement of the Toll pathway to resist bacteria, fungi, and parasitoids. This contributes to the idea that Spätzle may work more as a cytokine involved in general response to infection than to specific pathogens [5]. This Toll antiviral resistance is dependent on Dorsal and not Dif and we show Dorsal activation in virus-infected cells. The specificity of the immune response to difference pathogens may therefore rely on which transcription factors are activated downstream of the Toll pathway. Finally, we show that Toll requirement is restricted to viral oral infection and therefore route specific. This demonstrates that the interaction of viruses with *Drosophila* varies with mode of infection. Oral infection with viruses may be subject to more layers of control since it is probably the most frequent route of infection. Understanding this complexity is particularly relevant because arboviruses are transmitted to arthropod vectors of human diseases through feeding.

## Materials and Methods

### Fly strains and husbandry

Flies were maintained on standard cornmeal diet at a constant temperature of 25°C unless otherwise stated. All fly lines were cleaned of possible chronic viral infections as described elsewhere [22], [37]. Briefly, flies were aged to 30 days at 25°C and their eggs were collected in agar plates, treated with 50% bleach for 10 min, washed with water, and transferred to fresh vials.

Fly lines used in this study were free of *Wolbachia* except if otherwise stated. To mark midgut epithelial we used flies carrying the driver *Myo1A-Gal4* (expressed in the enterocytes [89]) combined with UAS-GFP. We have analysed the following homozygous or heterozygous combination of mutants in the Toll pathway: *spz^4^*/*spz^4^* (*spz^4^* is a loss of function allele) [90], *Tl^rv1^*/*Tl^r3^* (*Tl^rv1^* is a loss of function allele and *Tl^r3^* is a hypomorphic allele) [91], *pll^2^*/*pll^21^* (*pll^2^* is loss of function allele and *pll^21^* is a hypomorphic) [90], [92], *dl^1^*/*dl^1^*(*dl^1^* is a loss of function allele) [93], *Dif^1^*/*Dif^1^* (*Dif^1^* is a loss of function allele) [53]. To reduce genetic background effects these mutations were isogenized to the DrosDel *w^1118^* isogenic background [52]. For each line the non-mutated chromosomes were replaced using balancer chromosomes whereas the mutation was recombined to the respective DrosDel *w^1118^* isogenic chromosome for seven generations. We confirmed that the isogenized lines retained the mutation of interest by the associated development phenotype (lethality or maternal effect) or by DNA sequencing in the cases of absence of phenotype. For *Drosomycin* expression we used *y w drs-GFP dpt-LacZ* flies. For tissue specific *pll* knockdown the following drivers were used: *C7-Gal4* (fat body driver [94]), *24B-Gal4* (visceral muscle driver [95]), *Myo1A-Gal4* (midgut epithelium [89], [96]), *mef2-Gal4* (somatic, visceral and cardiac muscle [97]) and *hml(delta)-Gal4* (haemocyte driver [98]). *Tl^r3^* (#3238) and *dl^1^* (#3236) were obtained from the Bloomington stock center (http://flystocks.bio.indiana.edu/). Three independent *UAS-pll-IR* constructs and control *UAS-mCherry-IR* flies from TRiP collection [99] were used y^1^ sc^*^ v^1^; P{TRiP.HMS01213}attP2 (#34733), y^1^ sc^*^ v^1^; P{TRiP.GL00150}attP2 (#35577), y^1^ sc^*^ v^1^; P{TRiP.HMS02332}attP40 (#41935), y^1^ sc^*^ v^1^; P{VALIUM20-mCherry}attP2 (#35785). *MyoIA*-*Gal4* was kindly given by Nicolas Tapon, *spz^4^* and *y w drs-GFP dpt-LacZ* by Bruno Lemaitre, *Tl^rv1^* by Kathryn Anderson, *pll^2^* and *pll^21^* by Steven Wasserman and *Dif^1^* by Dominique Ferrandon.

### Virus production and titration

DCV was produced either in cell culture or in flies. Cell culture DCV production and titration were performed as described in [37]. DCV production in flies was done in *w^1118^ iso* flies that were clear from viruses and *Wolbachia* infection [37], [100]. Flies were afterwards orally infected with DCV, which led to the establishment of a chronically infected stock. This stock was kept for at least five generations before extracting DCV from it. Because DCV infected stocks show a high lethality rate at pupal stage, we perform DCV extraction from pupae. We squashed 50 g of pupae in 50 ml of 50 mM Tris-HCl, pH 7.5. The extract was frozen at −80°C, thawed and centrifuged twice for 20 min at 27000 *g* at 4°C, keeping the supernatant. The supernatant was aliquoted and stored at −80°C and later titrated in S*chneider's Line* 2 (SL-2) cells as described in [37]. FHV and CrPV was produced and titrated in Schneider Drosophila line 2 (DL2) as in [37] and in [101], respectively, with minor changes. Nora virus extract was prepared from a naturally infected Oregon R stock [37]. One hundred adult flies were squashed in 1 ml of 50 mM Tris-HCl, pH 7.5. Extract was then frozen at −80°C, thawed and twice centrifuged for 10 min at 20000 *g*, at 4°C. The supernatant was aliquoted and stored at −80°C.

### Viral infections and survival assays

Infections were performed on 3–6 days-old flies. To perform oral infection with virus we used empty plastic vials with 1×3 cm pieces of filter paper (Whatman gel blotting papper GB003) placed in the bottom. We loaded on the filter paper 350 µl of a mix of 75% virus extract and 25% of yeast (*Saccharomyces cerevisiae*, Sigma-Aldrich). Ten flies were placed per vial and left feeding for 24 hours at 25°C. For mock oral infections flies were exposed to buffer (50 mM Tris-HCl) mixed with yeast (25%). After this infection period we transferred the flies to new vials containing standard cornmeal diet. For viral systemic infections CO_2_ anesthetized flies were pricked in the thorax. The 0.15 mm diameter needles used for infection (Austerlitz Insect Pins) were dipped into a virus solution diluted to the desired concentration in 50 mM Tris-HCl, pH 7.5. After systemic infections flies were transferred to vials containing standard cornmeal food, 10 flies per vial. After both protocols of infection flies were kept at 25°C, checked for survival daily and vials changed every 5 days.

### Bacteria infection

*Pseudomonas entomophila* was grown in LB at 30°C overnight. *P. entomophila* cultures were then concentrated by centrifugation and adjusted to OD_600_ = 75. For oral infections with *P. entomophila* flies were exposed to a 1∶1 solution of bacteria culture and 5% sucrose in water. In control mock infections, flies were exposed to LB with 5% sucrose. Survival was followed every 12 hours for 3 days. *Micrococcus luteus* was grown in LB at 37°C overnight, concentrated by centrifugation and adjusted to OD_600_ = 3. For systemic infections with *M. luteus* flies were pricked in the thorax with fine needles dipped in bacterial suspension. The *P. entomophila* and *M. luteus* strains used in this study were kindly provided by Bruno Lemaitre and Thomas Rival, respectively.

### Immunostaining and microscopy

Flies were dissected to expose the internal tissues, fixed in 4% paraformaldehyde in phosphate-buffered saline (PBS) for 15 min, washed in PBS, then incubated with 1% Triton-X-100 and 5% FBS in PBS (PTX-FBS) for 30 min. Samples were then incubated overnight with primary antibody at 4°C. Rabbit polyclonal antibodies raised against purified DCV (kindly given by Peter Christian) was used at 1∶200 dilution. Dorsal antibody developed by Ruth Steward was obtained from the Developmental Studies Hybridoma Bank, created by the NICHD of the NIH and maintained at The University of Iowa, Department of Biology, Iowa City, IA 52242 [62], was used at 1∶5 dilution. The samples were washed with PTX-FBS, and then incubated in PTX-FBS with secondary antibodies conjugated with Alexa Fluor 488 or Alexa Fluor 568 (both by Molecular Probes) for 1 h. Samples were then washed with PTX-FBS, and incubated with Alexa Fluor 594 Phalloidin and DAPI or TOTO-3 (all by Molecular Probes) for 15 min. The samples were then washed in PTX-FBS, dissected and mounted in Vectashield Mounting Medium for microscopy. Confocal images were taken with either a Leica SP5 or Zeiss LSM 510 META confocal microscopes and processed in Fiji [102].

### Western blots

3–6 day old males of each line were orally infected with DCV (10^11^ TCID_50_/ml), collected 1, 3 and 5 days later for protein extraction, and probed in a Western blot with anti-DCV antibody. Ten males were pooled per sample. Rabbit polyclonal antibodies raised against purified DCV was kindly given by Dr. Peter Christian. E7 mouse monoclonal anti-*β*-tubulin was acquired from Developmental Studies Hybridoma Bank [103].

### RNA extractions and cDNA synthesis

For each sample RNA was extracted from one male fly using the Zymo Research Direct-zol RNA MiniPrep kit according to manufacturer's instructions. RNA concentrations were determined using *NanoDrop* ND-1000 Spectrophotometer. cDNA was prepared from 1 µg of total RNA using Random Primers and M-MLV Reverse Transcriptase (both Promega). Primers were allowed to bind to the template RNA for 5 min at 70°C and the reaction proceeded at 25°C for 10 min, 37°C for 60 min and 80°C for 10 min.

### Real-time quantitative PCR

Each cDNA sample was analyzed in triplicate using a 7900HT Fast Real-Time PCR System (Applied Biosystems) instrument. We performed each reaction in a 384-well plate (Applied Biosystems), using 7 µl of iQ SYBR Green supermix (Bio Rad), 0,5 µl of each primer solution at 3,6 µM and 5 µl of diluted cDNA. Viral amplification was performed using the following thermal cycling protocol: initial 50°C for 2 min, denaturation for 10 min at 95°C followed by 40 cycles of 30 s at 95°C, 1 min at 56°C and 30 s s at 72°C. Melting curves were analysed to confirm specificity of amplified products. We obtained Ct values for manual threshold of 10 using the program SDS 2.4. Relative amounts of viral RNA were calculated by the Pfaffl Method [104] using *Drosophila Rpl32* as a reference gene. The following primers were used: DCV forward 5′- TCATCGGTATGCACATTGCT-3′; DCV reverse 5′-CGCATAACCATGCTCTTCTG-3′; FHV forward 5′- ACCTCGATGGCAGGGTTT-3′; FHV reverse 5′- CTTGAACCATGGCCTTTTG-3′; CrPV forward 5′-ACGAGGAAGCAACTCAAGGA-3′; CrPV reverse 5′-GAGCCCGCTGAGATGTAAAG-3′; Nora forward 5′-TTTCACTTTACTGTTGGTCTCC-3′; Nora reverse 5′-ATTCCATTTGTGACTGATTTTATTTC-3′; Rpl32 forward 5′- CCGCTTCAAGGGACAGTATC-3′; Rpl32 reverse 5′-CAATCTCCTTGCGCTTCTTG-3′.

### Germ-free like conditions

Flies *w^1118^ iso* and *pll* ^−/−^ were raised for one generation in food with a mix of antibiotics (100 µg/ml of streptomycin, 200 µg/ml of rifampicin and 100 µg/ml of tetracycline) [79], [105] and progeny was used to test susceptibility to virus. Flies were maintained in antibiotic food until the end of survival analysis. Elimination of bacteria was confirmed by plating homogenates of *pll* ^−/−^ flies that died during the time-course of infection. For each condition, a pool of 3 dead flies was homogenized with a pestle in 100 µl of LB. The homogenized extract was plated with the help of a 10 µl inoculation loop in Lactobacilli MRS broth and Mannitol culture media, which are able to grow *Lactobacillus* and *Acetobacter*, respectively [106]. The plates were incubated for 4 days at 25°C and subsequently scanned.

### Statistics

All statistical analyses were done using R (2.10.1) [107].

To compare survival rates we used a Cox's proportional hazards mixed effect model (*coxme* in R). Fixed effects include sex, viral dose, genotype, presence/absence of *Wolbachia*, antibiotic treatment, and repeat of the experiment. To account for variation between vials of the same line in the same experiment, replicate vials were considered as a random effect. This method accounts for variation between vials of the same line in the same experiment and variation between replicates of the experiment.

To assess the significance of the different fixed factors and their interactions, we performed stepwise backward model selection, and compared the difference in the log-likelihood of the different models with a *χ^2^* distribution, with the appropriate degrees of freedom.

To compare the different doses or the different genotypes with each other we performed either pairwise comparisons between all levels of the factors (Tukey-like contrasts [108]) or contrasted the genotype of interest with the respective genetic backgrounds, averaging for the effect of the remaining factors. When the interaction between factors was statistically significant, the factor of interest was compared independently for the different levels of the interacting variable. When needed, and in order to obtain independent estimates of the hazard ratios (e.g. between different genotypes, with and without infection), we calculated the hazard ratios in models which included the interaction term, despite the interaction being non-significant. Multiple comparisons were performed using the “multcomp” (function *glht*) and “lsmeans” (function *lsmeans*) packages in R.

In order to compare viral loads between genotypes we used a Wilcoxon rank sum test (*wilcox.test* in R).

To analyze the feeding rates we used a generalized linear model (GLM) with a binomial response, with the proportion of fed versus unfed flies as a dependent variable and genotype and time as fixed factors.

The *p*-value of the chi-square test (*chisq.test* in R) was computed for a Monte Carlo test with 10^9^ replicates.

## Supporting Information

Figure S1 **Cox hazard ratios of DCV orally infected** ***w^1118^ iso*** **flies.** Cox hazard ratios of DCV orally infected *iso w^1118^* flies compared with mock treatment at different concentrations. Letters refer to statistically homogenous groups of hazards, based on Tukey's pairwise comparisons between all treatments. All DCV treatments had significantly higher mean hazard when compared with mock infection (*p*<0.001 in all cases), which was assigned group “d” (not shown). Natural logarithm of Cox hazard ratio is shown and error bars represent standard error. The analysis is based on three independent experiments of males and females, each with 60 flies per line, with 10 flies per vial.
(TIF)

Figure S2 **DCV antibody specificity.** (A–F) Adult male tissues were dissected and immunostained with antibody against DCV after mock oral infection. (A–E) DCV was immunostained with an antibody (green), actin marked with phalloidin (red) and DNA marked with TOTO3 (blue). (F) Haemocytes were marked with GFP expression (green) driven by *hml(delta)-Gal4*, DCV was immunostained with an antibody (red), and DNA marked by DAPI (blue). All experiments were performed in flies 3–6 days old.
(TIF)

Figure S3 **DCV tissue tropism upon oral infection.** (A) DCV infection in the fat body. (B) Malpighian tubules are not infected with DCV, but the muscle cells surrounding the Malpighian tubules near the junction with the gut are infected. MT - Malpighian tubules, MG - Midgut. (C) Abdominal muscles infected with DCV. (D) Thoracic muscles not infected with DCV. (E) Trachea infected with DCV. (A–E) DCV was immunostained with an antibody (green), actin marked with phalloidin (red) and DNA marked with TOTO3 (blue). All tissues were dissected from adult flies 5 dpi. DCV infections (10^11^ TCID_50_/ml) were performed in 3–6 days old flies.
(TIF)

Figure S4 **Cox hazard ratios of Toll pathway mutant flies upon DCV oral infection.** (A) Cox hazard ratio of Toll pathway mutant lines compared to *w^1118^ iso* when orally infected with DCV (10^11^ TCID50/ml). (B) Cox hazard ratio of *Tl* mutant flies compared to *w^1118^ iso* when orally infected with DCV (10^11^ TCID50/ml) (*p*<0.001). (C) Cox hazard ratio of Toll pathway mutant lines compared to *w^1118^ iso* when mock orally infected. (A–C) The natural logarithm of Cox hazard ratio is shown and error bars represent standard error. (A,C) Letters refer to statistically homogenous groups of hazards, based on Tukey's pairwise comparisons between all treatments, *w^1118^ iso* is assigned to group “a” (not shown). Survival assays for oral infections were performed thrice for *pll*, *spz*, an *dl* mutants, and twice for *Dif* and *Tl* mutants, each with 60 flies per line, with 10 flies per vial.
(TIF)

Figure S5 ***pll*** **mutants sensitivity to DCV oral infections.** (A–D) Survival of *pll^−/−^* and *w^1118^ iso* to different doses of DCV oral infection (A at 10^9^, B at 10^10^, C at 10^11^ TCID_50_/ml and D mock). For all DCV doses *pll^−/−^* mutant flies were more susceptible to DCV oral infection than *w^1118^ iso* control flies (Cox Proportional Hazards Model, *p* = 0.023, *p*<0.001 and *p*<0.001 respectively). For all survival experiments, sixty 3–6 days old males, per line, were infected orally with DCV or buffer, and their survival was monitored daily. (E) DCV RNA levels 5 days after oral infection (10^11^ TCID_50_/ml). DCV loads are significantly different between *pll^−/−^* and *w^1118^ iso* line (Wilcoxon test, *p*<0.001).
(TIF)

Figure S6 **DCV protein levels after oral infection.** 3–6 days old males of *pll^−/−^* and *w^1118^ iso* lines were orally infected with DCV (10^11^ TCID_50_/ml), collected 1,3 or 5 days later for protein extraction, and probed in a Western blot with anti-DCV antibody (10 flies per sample). *pll^−/−^* flies mock infected were used as control. Anti-tubulin antibody was used as a loading control.
(TIF)

Figure S7 **Toll pathway mutant flies are not more sensitive to DCV systemic infections.** (A) Cox hazard ratios of Toll pathway mutant lines compared to *w^1118^ iso* when systemically infected with DCV (10^7^ TCID50/ml). None of the mutant lines were significantly different from *w^1118^ iso* (Cox proportional hazard mixed effect model, *p*>0.1). (B) Survival of Toll pathway mutant lines upon pricking with buffer only. Sixty 3–6 days old males of each line were pricked and their survival was monitored daily. (C) Cox hazard ratios of Toll pathway mutant line compared to *w^1118^ iso* when pricked with buffer only (mock). None of the mutant lines were significantly different from *w^1118^ iso* (Cox proportional hazard mixed effect model, *p*>0.09). (A and C) The natural logarithm of Cox hazard ratio is shown and error bars represent standard error. Survival data of two experiments was analysed together. Each experiment had 60 flies per line, with 10 flies per vial. Letters refer to statistically homogenous groups of hazards, based on Tukey's pairwise comparisons between all treatments. *w^1118^ iso* is assigned to group “a” in the compact letter display of Tukey's test (not shown).
(TIF)

Figure S8 **Lack of interaction between** ***Drosophila*** **-associated bacteria and Toll pathway protection to viruses.** (A–C) *Wolbachia* protection to DCV systemic infection does not require the Toll pathway. Sixty 3–6 days old males of each line were pricked with DCV at 10^6^ TCID_50_/ml (A), 10^7^ TCID_50_/ml (B) or mock (C), and the survival was monitored daily. Survival data of both doses was fitted together with a Cox proportional hazard mixed effect model. There is no interaction between *Wolbachia* and genotype (*p* = 0.73). (D) Demonstration of germ-free-like conditions using antibiotic treated food. Flies raised in antibiotic treated food (left side of plates) or control food (right side of plates) were homogenized and plated in Lactobacilli MRS broth (D) or in Mannitol broth (E) agar culture media. (F) Cox hazard ratios of antibiotic-treated *w^1118^ iso* and conventionally reared or antibiotic-treated *pll^−/−^* flies, with conventionally reared *w^1118^ iso* flies, after oral infection with DCV. Natural logarithm of Cox hazard ratio is shown and error bars represent standard error. Letters refer to statistically homogenous groups of hazards ratios, based on Tukey's pairwise comparisons between all genotypes and antibiotic treatment combinations. Either with or without antibiotic treatment, *pll^−/−^* flies had significantly higher mean hazard compared with *w^1118^ iso* flies (*p*<0.001 in both cases), which was assigned group “a” (not shown). In both genotypes, antibiotic treated flies showed no differences in survival, compared with conventionally reared flies (*p* = 0.97 and *p* = 0.96 for the comparison between conventionally reared and antibiotic treated, in the *w^1118^ iso* and *pll^−/−^* flies, respectively). The analysis is on 60 males per line, with 10 flies per vial.
(TIF)

Figure S9 **Cox hazard ratios of** ***pll^−/−^*** **and** ***w^1118^ iso*** **lines after CrPV, Nora and FHV infection.** Cox hazard ratios of *pll^−/−^* mutant lines compared to *w^1118^ iso* when (A) orally infected with CrPV (1.76×10^10^ TCID50/ml); (B) systemically infected with CrPV at 10^6^, 10^7^ and 10^8^ TCID50/ml; (C) orally infected with Nora virus; (D) orally infected with FHV (10^10^ TCID50/ml); (E) systemically infected with FHV at 10^6^, 10^7^ and 10^8^ TCID50/ml. *pll^−/−^* mutants showed a significantly increased hazard relative to *w^1118^ iso* after oral infection with CrPV and FHV (Cox proportional hazard mixed effect model, *p*<0.001 in both cases). After oral infection with Nora virus or systemic infection with different doses of CrPV or FHV there were no statistically significant differences between the genotypes (Cox proportional hazard mixed effect model, *p*≥0.25 for all comparisons). (A–E) Survival analysis based on one (B and E) or three independent experiments (A, C, D), each with 60 flies per line, with 10 flies per vial. The natural logarithm of Cox hazard ratio is shown and error bars represent standard error. In panels B and E *pll^−/−^* survival at each dose is compared with *w^1118^ iso* infected at the corresponding dose.
(TIF)

Figure S10 ***pll*** **mutant and** ***w^1118^ iso*** **flies have similar sensitivity to** ***Pseudomonas entomophila*** **oral infection and similar ingestion rates.** (A) Sixty 3–6 days-old males *pll^−/−^* and *w^1118^ iso* were orally infected with *Pseudomonas entomophila* (75 OD) or buffer, and the survival was checked twice a day. Survival data was fitted with a Cox proportional hazard mixed effect model. *pll^−/−^* is not significantly different from *w^1118^ iso* (*p* = 0.303). (B) 3–6 days-old *pll^−/−^* and *w^1118^ iso* males, were exposed to DCV mixed with yeast supplemented with 0,1% bromophenol blue solution. Ingestion rates were measured after 15 min, 30 min, 1 h, 2 h and 24 h by counting flies that had blue abdomens under a dissection microscope. Fifty males per time point were used. Data was fitted with a general linear model. *pll^−/−^* mutant and *w^1118^ iso* ingestion rates are not different (*p* = 0.626).
(TIF)

Figure S11 **Subcellular localization of Dorsal in fat body and midgut.** (A) Lack of Dorsal nuclear import in fat body 5 days after mock oral infection. 6 flies were analysed (B) Absence of Dorsal staining in fat body cells of *dl^−/−^* (*dl^1^/dl^1^*) mutant flies, 5 days after mock oral infection. (A–B) Adult male fat body was immunostained with antibody against Dorsal (green), actin marked with phalloidin (red), and DNA marked with DAPI (blue). (C) Midgut muscle cells infected with DCV 5 days after oral infection showing no nuclear import of Dorsal. (D) Midgut epithelial cells 5 days after oral infection showing no nuclear import of Dorsal. (C–D) 14 DCV-positive adult males were analysed, guts were immunostained with an antibody against Dorsal (green), an antibody against DCV (red), actin marked with phalloidin (white) and DNA was marked with DAPI (blue). DCV was at 10^11^ TCID_50_/ml. (E) Lack of Dorsal nuclear import in haemocytes 5 days after DCV oral infection. Adult male haemocytes were immunostained with an antibody against Dorsal (green), an antibody against DCV (red) and DNA was marked with DAPI (blue). (F–G) *Drs*-GFP expression in muscle (F) and epithelium (G) of midgut after 5 days DCV oral infection. (F–G) Adult male midguts were immunostained with antibody against DCV (red), antibody against GFP (green) and DNA marked with TOTO3 (blue). (C–G) DCV dose was 10^11^ TCID_50_/ml.
(TIF)

Figure S12 **Tissue specific expression of** ***pll*** **RNAi constructs has no effect on survival against oral DCV infection.** Survival of three independent *UAS-pll-IR* constructs and control *UAS-mCherry-IR* flies upon DCV oral infection (10^11^ TCID_50_/ml) or buffer, using tissue specific drivers. Tissue specific *UAS-pll-IR* expression lines were not more sensitive than control lines, using any of the tested constructs. (**A**) Fat body specific *pll-IR* expression using *C7-Gal4* (Genotype effect, Cox proportional hazard mixed effect model, *p* = 0.35). (**B**) Visceral muscle specific *pll-IR* expression using *24B-Gal4* driver (Genotype effect, Cox proportional hazard mixed effect model, *p* = 0.39). (**C**) Midgut epithelium specific *pll-IR* expression using *Myo1A-Gal4* (Genotype effect, Cox proportional hazard mixed effect model, *p* = 0.51). (**D**) Haemocyte specific *pll-IR* expression using *hml(delta)-Gal4* (Genotype effect, Cox proportional hazard mixed effect model, *p* = 0.12). (**E**) Somatic, visceral and cardiac muscle specific *pll-IR* expression using *mef2-Gal4* (Genotype effect, Cox proportional hazard mixed effect model, *p*<0.01; multiple comparisons between *UAS-pll-IR* lines and UAS-*mCherry*-*IR*-line, p>0.43). For all experiments, sixty 3–6 days old males, per line and condition were used, with 10 flies per vial. Flies were orally infected with DCV or buffer, and their survival was monitored daily. Each survival assay was performed twice. Survival data of both replicates was analysed together using Cox proportional hazard mixed effect models.
(TIF)

Dataset S1 **Survival of adult** ***w^1118^ iso*** **flies after DCV oral infection.**
(CSV)

Dataset S2 **Survival of Toll pathway mutants after DCV oral infection.**
(CSV)

Dataset S3 **Survival of Toll gene mutant after DCV oral infection.**
(CSV)

Dataset S4 **Survival of Toll pathway mutants after mock oral infection.**
(CSV)

Dataset S5 **Survival of** ***pll^−/−^*** **and** ***w^1118^ iso*** **flies to different doses of DCV oral infection.**
(CSV)

Dataset S6 **DCV RNA levels in** ***pll^−/−^*** **and** ***w^1118^ iso*** **flies after oral infection, at 2, 5, 10 and 20 dpi.**
(CSV)

Dataset S7 **Survival of Toll pathway mutants after DCV systemic infection.**
(CSV)

Dataset S8 **Survival of Toll pathway mutants after mock systemic infection.**
(CSV)

Dataset S9 **Survival of** ***pll^−/−^*** **and** ***w^1118^ iso*** **flies to different doses of DCV systemic infection.**
(CSV)

Dataset S10 **Survival of** ***pll^−/−^*** **and** ***w^1118^*** **iso with and without** ***Wolbachia*** **after DCV oral infection.**
(CSV)

Dataset S11 **Survival of** ***pll^−/−^*** **and** ***w^1118^*** **iso with and without** ***Wolbachia*** **after DCV systemic infection.**
(CSV)

Dataset S12 **Survival of conventionally reared and antibiotic treated** ***pll^−/−^*** **and** ***w^1118^ iso*** **flies after DCV oral infection.**
(CSV)

Dataset S13 **Survival of** ***pll^−/−^*** **and** ***w^1118^ iso*** **flies after CrPV oral infection.**
(CSV)

Dataset S14 **DCV, CrPV, Nora and FHV RNA levels in** ***pll^−/−^*** **and** ***w^1118^ iso*** **flies 5 days after oral infection.**
(CSV)

Dataset S15 **Survival of** ***pll^−/−^*** **and** ***w^1118^ iso*** **flies to different doses of CrPV systemic infection.**
(CSV)

Dataset S16 **Survival of** ***pll^−/−^*** **and** ***w^1118^ iso*** **flies after Nora oral infection.**
(CSV)

Dataset S17 **Survival of** ***pll^−/−^*** **and** ***w^1118^ iso*** **flies after FHV oral infection.**
(CSV)

Dataset S18 **Survival of** ***pll^−/−^*** **and** ***w^1118^ iso*** **flies to different doses of FHV systemic infection.**
(CSV)

Dataset S19 **Survival of** ***pll^−/−^*** **and** ***w^1118^ iso*** **flies after** ***Pseudomonas entomophila*** **oral infection.**
(CSV)

Dataset S20 **Feeding rates of** ***pll^−/−^*** **and** ***w^1118^ iso*** **flies.**
(CSV)

Dataset S21 **Survival of flies with tissue specific** ***pll^−/−^*** **knockdown after DCV oral infection.**
(CSV)
