# Supplementary figures and images for: The Toll-Dorsal Pathway Is Required for Resistance to Viral Oral Infection in Drosophila
Source: PLoS Pathog. 2014 Dec 4;10(12):e1004507. doi: 10.1371/journal.ppat.1004507 (PMC4256459; doi:10.1371/journal.ppat.1004507)

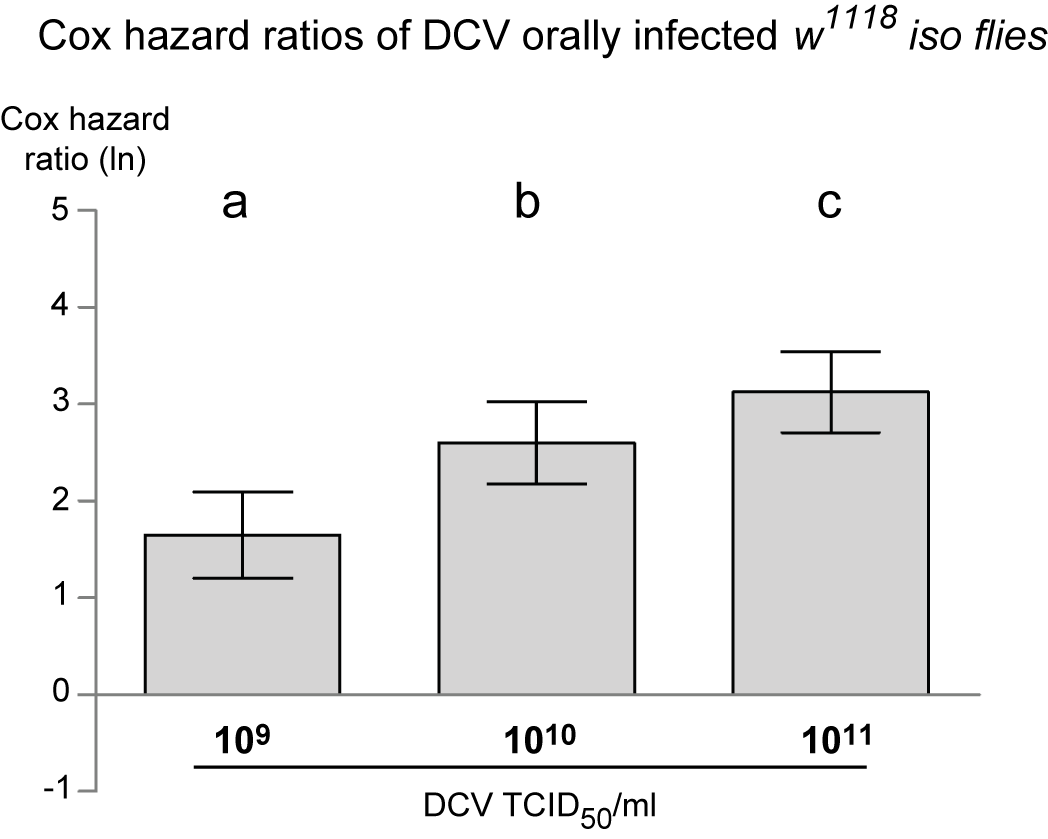

Supplement: Figure S1 — Cox hazard ratios of DCV orally infected w1118 iso flies. Cox hazard ratios of DCV orally infected iso w1118 flies compared with mock treatment at different concentrations. Letters refer to statistically homogenous groups of hazards, based on Tukey's pairwise comparisons between all treatments. All DCV treatments had significantly higher mean hazard when compared with mock infection (p<0.001 in all cases), which was assigned group “d” (not shown). Natural logarithm of Cox hazard ratio is shown and error bars represent standard error. The analysis is based on three independent experiments of males and females, each with 60 flies per line, with 10 flies per vial. (TIF) [file ppat.1004507.s001.tif]

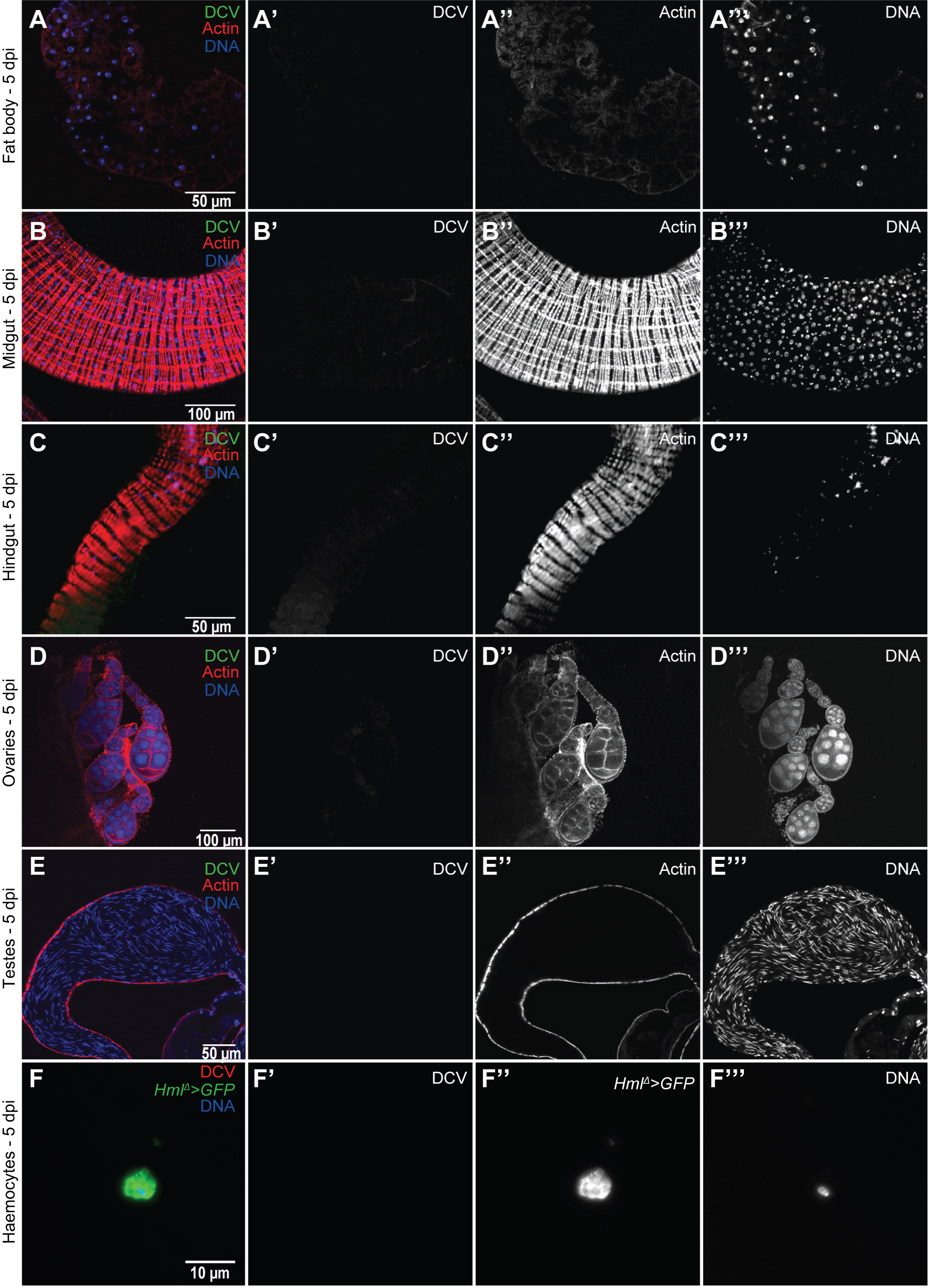

Supplement: Figure S2 — DCV antibody specificity. (A–F) Adult male tissues were dissected and immunostained with antibody against DCV after mock oral infection. (A–E) DCV was immunostained with an antibody (green), actin marked with phalloidin (red) and DNA marked with TOTO3 (blue). (F) Haemocytes were marked with GFP expression (green) driven by hml(delta)-Gal4, DCV was immunostained with an antibody (red), and DNA marked by DAPI (blue). All experiments were performed in flies 3–6 days old. (TIF) [file ppat.1004507.s002.tif]

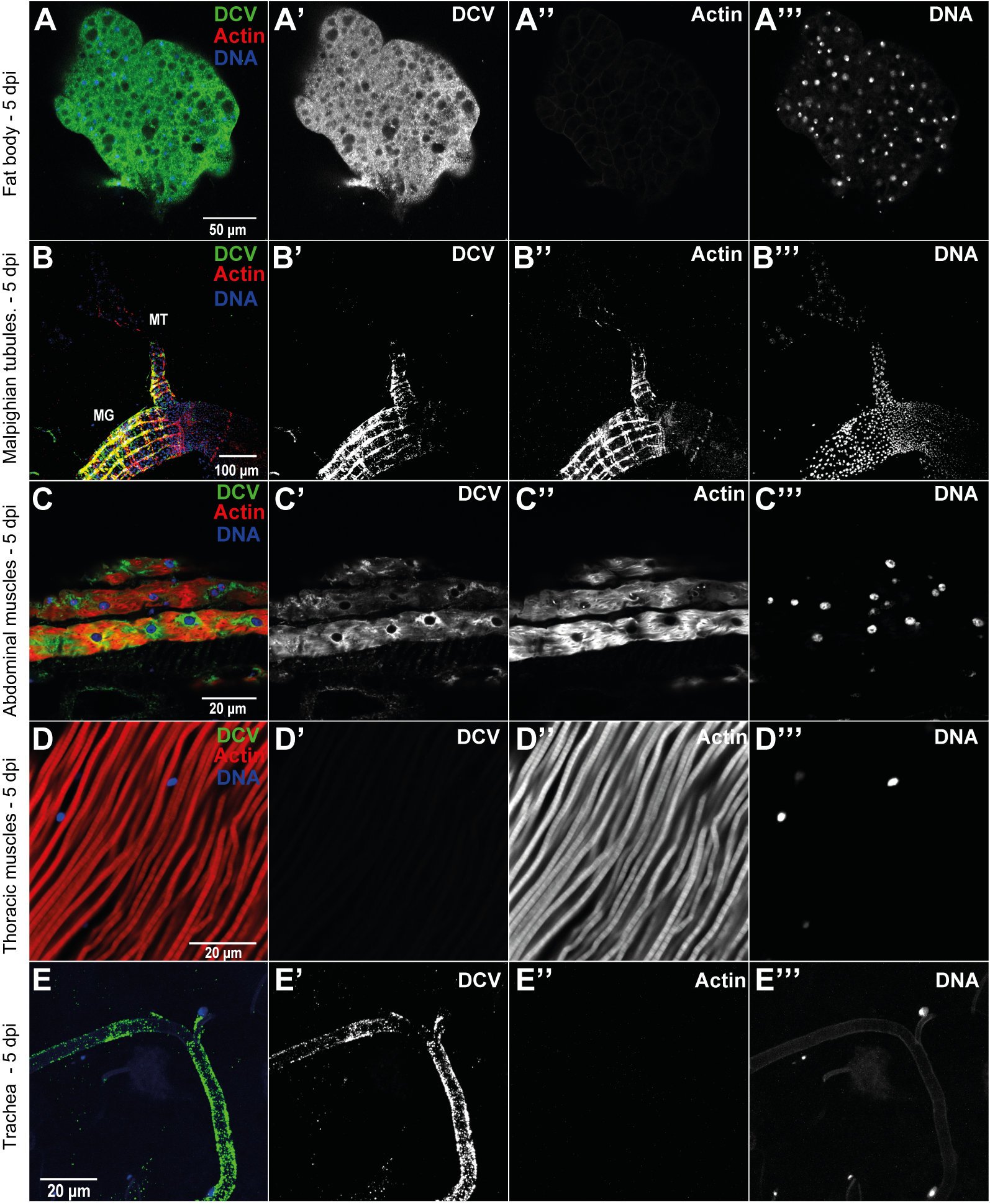

Supplement: Figure S3 — DCV tissue tropism upon oral infection. (A) DCV infection in the fat body. (B) Malpighian tubules are not infected with DCV, but the muscle cells surrounding the Malpighian tubules near the junction with the gut are infected. MT - Malpighian tubules, MG - Midgut. (C) Abdominal muscles infected with DCV. (D) Thoracic muscles not infected with DCV. (E) Trachea infected with DCV. (A–E) DCV was immunostained with an antibody (green), actin marked with phalloidin (red) and DNA marked with TOTO3 (blue). All tissues were dissected from adult flies 5 dpi. DCV infections (1011 TCID50/ml) were performed in 3–6 days old flies. (TIF) [file ppat.1004507.s003.tif]

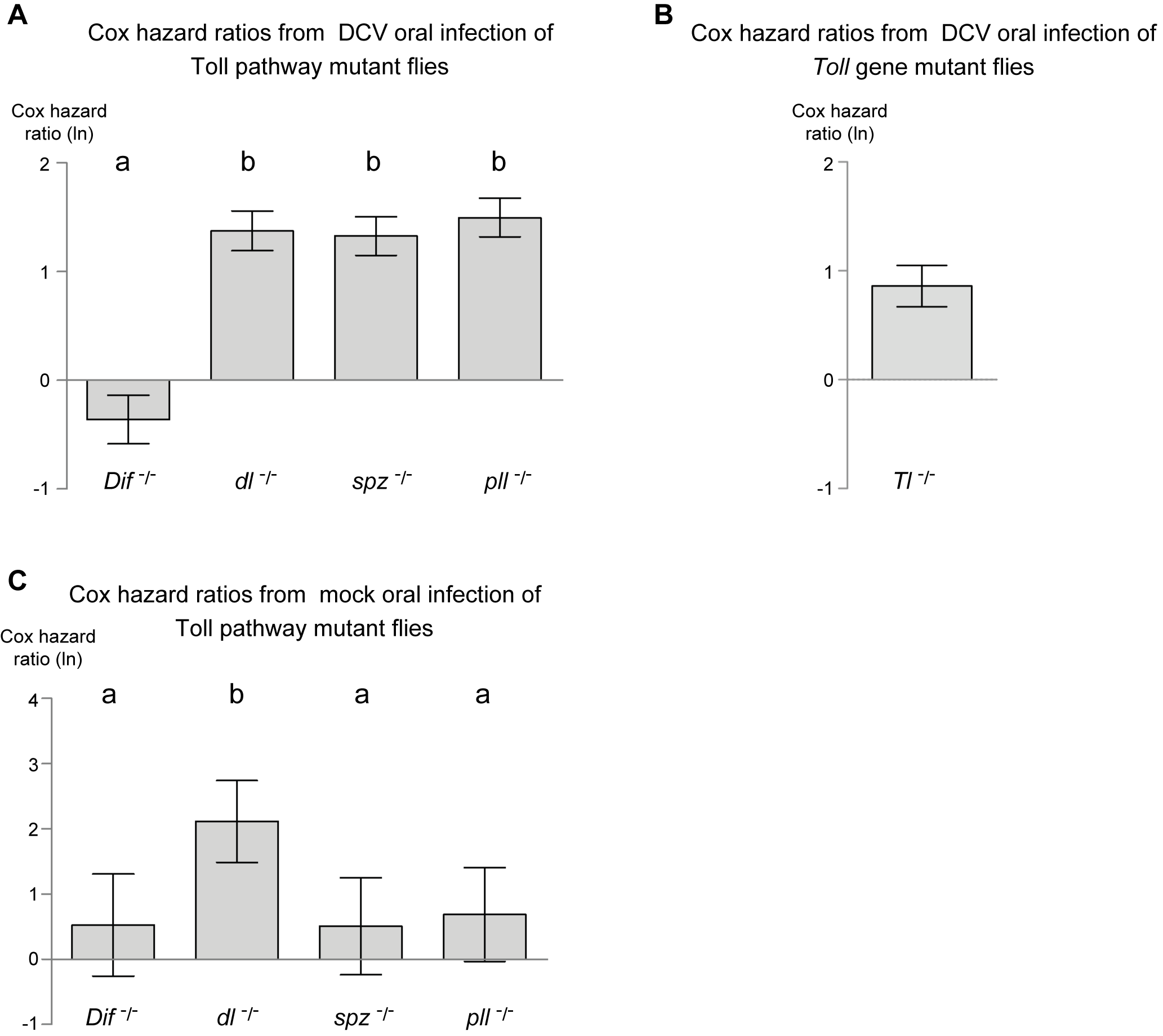

Supplement: Figure S4 — Cox hazard ratios of Toll pathway mutant flies upon DCV oral infection. (A) Cox hazard ratio of Toll pathway mutant lines compared to w1118 iso when orally infected with DCV (1011 TCID50/ml). (B) Cox hazard ratio of Tl mutant flies compared to w1118 iso when orally infected with DCV (1011 TCID50/ml) (p<0.001). (C) Cox hazard ratio of Toll pathway mutant lines compared to w1118 iso when mock orally infected. (A–C) The natural logarithm of Cox hazard ratio is shown and error bars represent standard error. (A,C) Letters refer to statistically homogenous groups of hazards, based on Tukey's pairwise comparisons between all treatments, w1118 iso is assigned to group “a” (not shown). Survival assays for oral infections were performed thrice for pll, spz, an dl mutants, and twice for Dif and Tl mutants, each with 60 flies per line, with 10 flies per vial. (TIF) [file ppat.1004507.s004.tif]

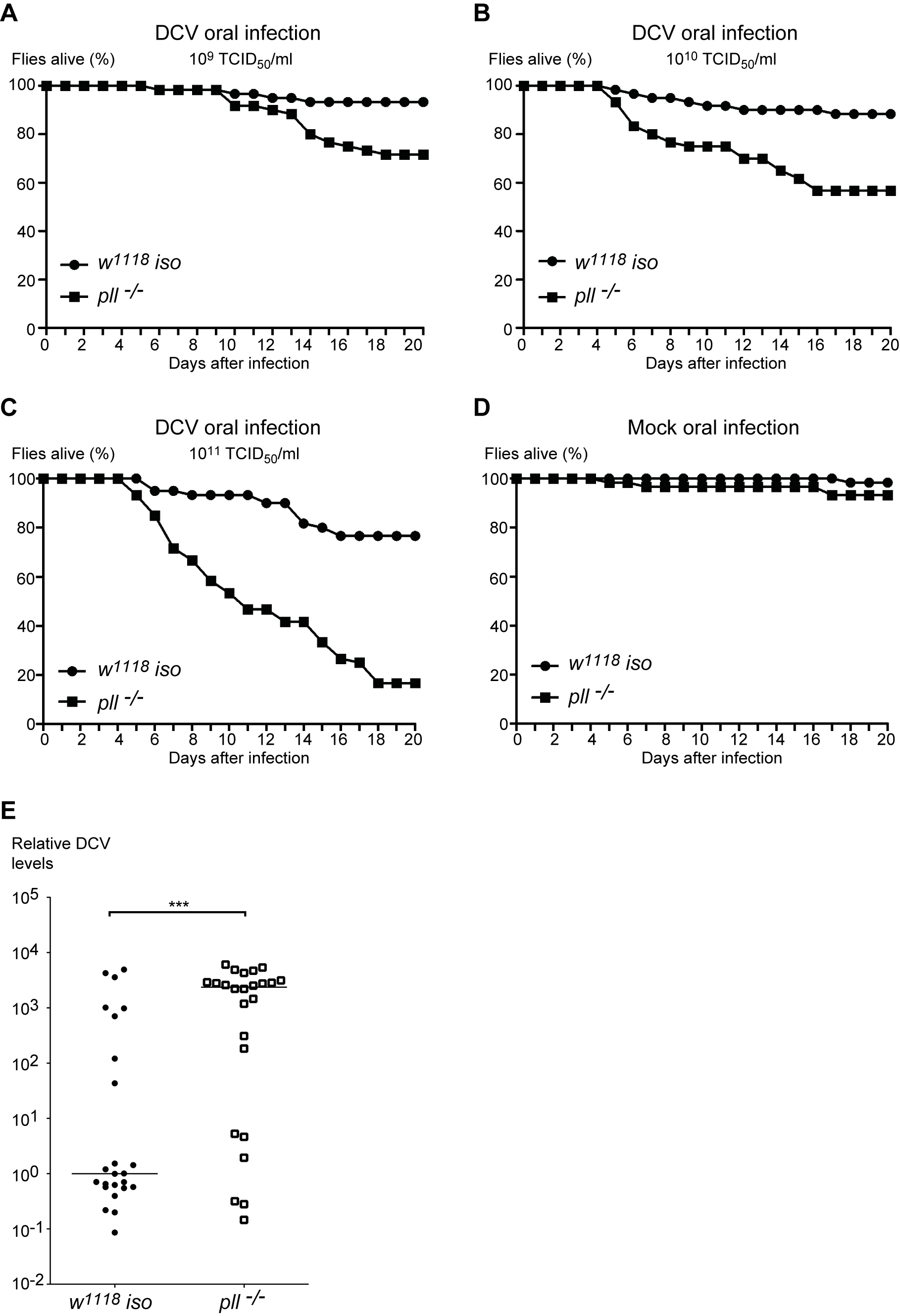

Supplement: Figure S5 — pll mutants sensitivity to DCV oral infections. (A–D) Survival of pll−/− and w1118 iso to different doses of DCV oral infection (A at 109, B at 1010, C at 1011 TCID50/ml and D mock). For all DCV doses pll−/− mutant flies were more susceptible to DCV oral infection than w1118 iso control flies (Cox Proportional Hazards Model, p = 0.023, p<0.001 and p<0.001 respectively). For all survival experiments, sixty 3–6 days old males, per line, were infected orally with DCV or buffer, and their survival was monitored daily. (E) DCV RNA levels 5 days after oral infection (1011 TCID50/ml). DCV loads are significantly different between pll−/− and w1118 iso line (Wilcoxon test, p<0.001). (TIF) [file ppat.1004507.s005.tif]

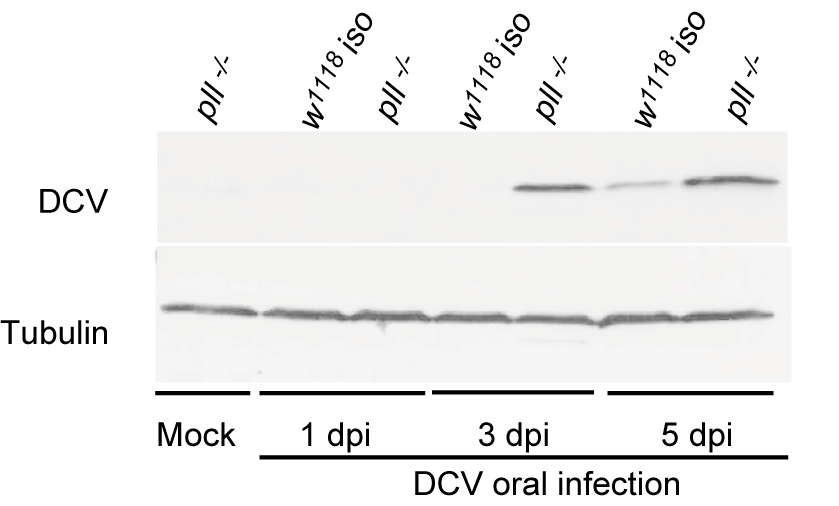

Supplement: Figure S6 — DCV protein levels after oral infection. 3–6 days old males of pll−/− and w1118 iso lines were orally infected with DCV (1011 TCID50/ml), collected 1,3 or 5 days later for protein extraction, and probed in a Western blot with anti-DCV antibody (10 flies per sample). pll−/− flies mock infected were used as control. Anti-tubulin antibody was used as a loading control. (TIF) [file ppat.1004507.s006.tif]

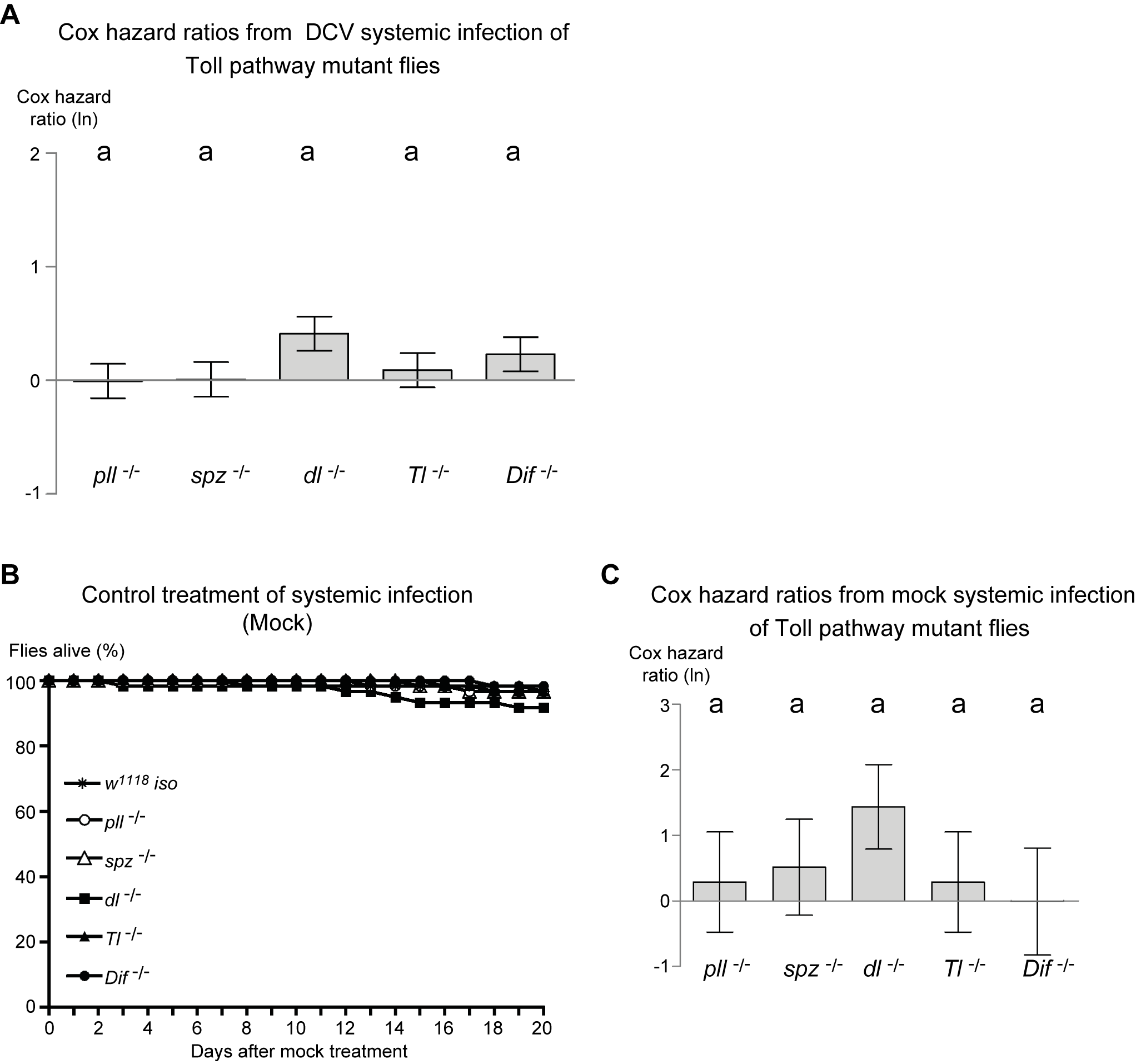

Supplement: Figure S7 — Toll pathway mutant flies are not more sensitive to DCV systemic infections. (A) Cox hazard ratios of Toll pathway mutant lines compared to w1118 iso when systemically infected with DCV (107 TCID50/ml). None of the mutant lines were significantly different from w1118 iso (Cox proportional hazard mixed effect model, p>0.1). (B) Survival of Toll pathway mutant lines upon pricking with buffer only. Sixty 3–6 days old males of each line were pricked and their survival was monitored daily. (C) Cox hazard ratios of Toll pathway mutant line compared to w1118 iso when pricked with buffer only (mock). None of the mutant lines were significantly different from w1118 iso (Cox proportional hazard mixed effect model, p>0.09). (A and C) The natural logarithm of Cox hazard ratio is shown and error bars represent standard error. Survival data of two experiments was analysed together. Each experiment had 60 flies per line, with 10 flies per vial. Letters refer to statistically homogenous groups of hazards, based on Tukey's pairwise comparisons between all treatments. w1118 iso is assigned to group “a” in the compact letter display of Tukey's test (not shown). (TIF) [file ppat.1004507.s007.tif]

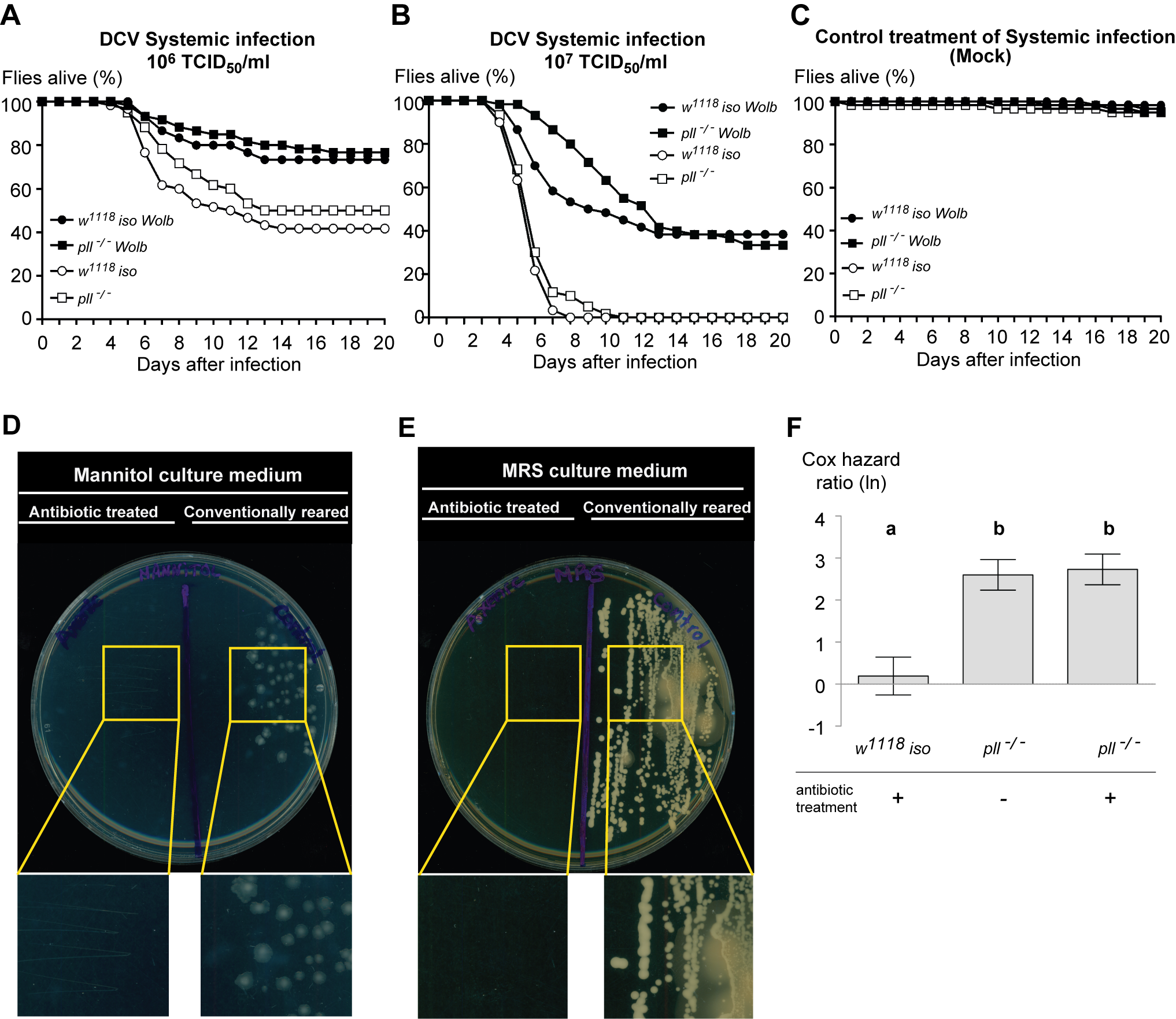

Supplement: Figure S8 — Lack of interaction between Drosophila -associated bacteria and Toll pathway protection to viruses. (A–C) Wolbachia protection to DCV systemic infection does not require the Toll pathway. Sixty 3–6 days old males of each line were pricked with DCV at 106 TCID50/ml (A), 107 TCID50/ml (B) or mock (C), and the survival was monitored daily. Survival data of both doses was fitted together with a Cox proportional hazard mixed effect model. There is no interaction between Wolbachia and genotype (p = 0.73). (D) Demonstration of germ-free-like conditions using antibiotic treated food. Flies raised in antibiotic treated food (left side of plates) or control food (right side of plates) were homogenized and plated in Lactobacilli MRS broth (D) or in Mannitol broth (E) agar culture media. (F) Cox hazard ratios of antibiotic-treated w1118 iso and conventionally reared or antibiotic-treated pll−/− flies, with conventionally reared w1118 iso flies, after oral infection with DCV. Natural logarithm of Cox hazard ratio is shown and error bars represent standard error. Letters refer to statistically homogenous groups of hazards ratios, based on Tukey's pairwise comparisons between all genotypes and antibiotic treatment combinations. Either with or without antibiotic treatment, pll−/− flies had significantly higher mean hazard compared with w1118 iso flies (p<0.001 in both cases), which was assigned group “a” (not shown). In both genotypes, antibiotic treated flies showed no differences in survival, compared with conventionally reared flies (p = 0.97 and p = 0.96 for the comparison between conventionally reared and antibiotic treated, in the w1118 iso and pll−/− flies, respectively). The analysis is on 60 males per line, with 10 flies per vial. (TIF) [file ppat.1004507.s008.tif]

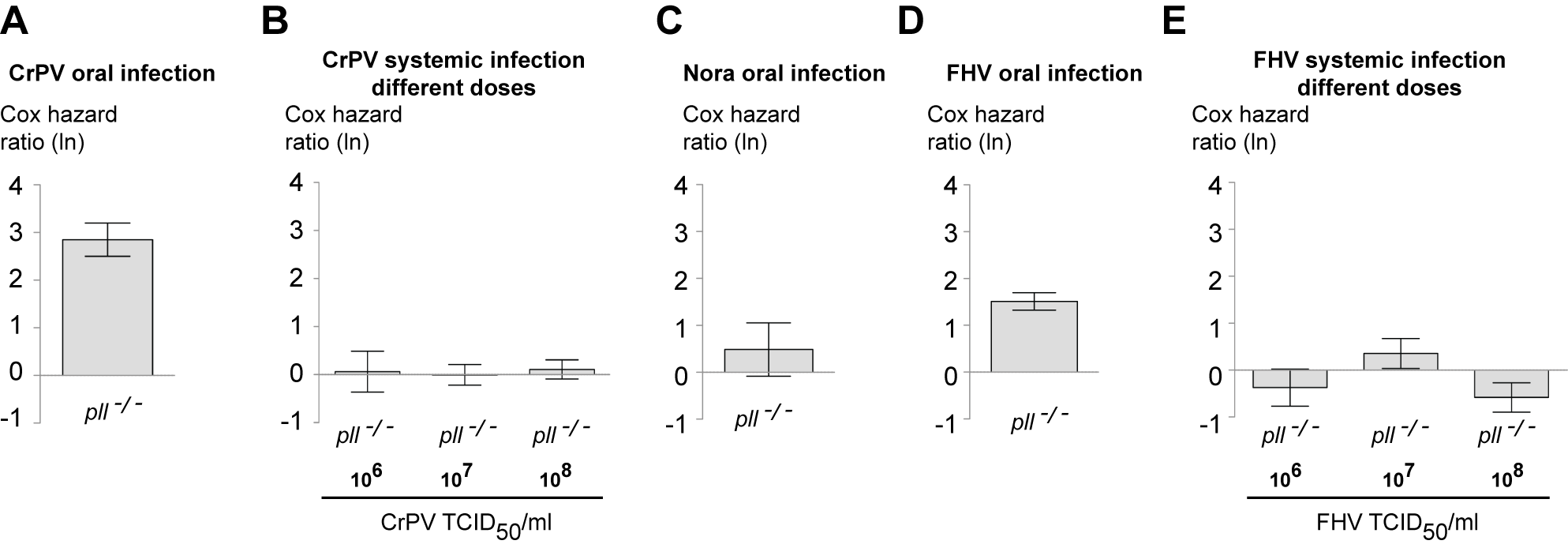

Supplement: Figure S9 — Cox hazard ratios of pll−/− and w1118 iso lines after CrPV, Nora and FHV infection. Cox hazard ratios of pll−/− mutant lines compared to w1118 iso when (A) orally infected with CrPV (1.76×10^10^ TCID50/ml); (B) systemically infected with CrPV at 106, 107 and 108 TCID50/ml; (C) orally infected with Nora virus; (D) orally infected with FHV (1010 TCID50/ml); (E) systemically infected with FHV at 106, 107 and 108 TCID50/ml. pll−/− mutants showed a significantly increased hazard relative to w1118 iso after oral infection with CrPV and FHV (Cox proportional hazard mixed effect model, p<0.001 in both cases). After oral infection with Nora virus or systemic infection with different doses of CrPV or FHV there were no statistically significant differences between the genotypes (Cox proportional hazard mixed effect model, p≥0.25 for all comparisons). (A–E) Survival analysis based on one (B and E) or three independent experiments (A, C, D), each with 60 flies per line, with 10 flies per vial. The natural logarithm of Cox hazard ratio is shown and error bars represent standard error. In panels B and E pll−/− survival at each dose is compared with w1118 iso infected at the corresponding dose. (TIF) [file ppat.1004507.s009.tif]

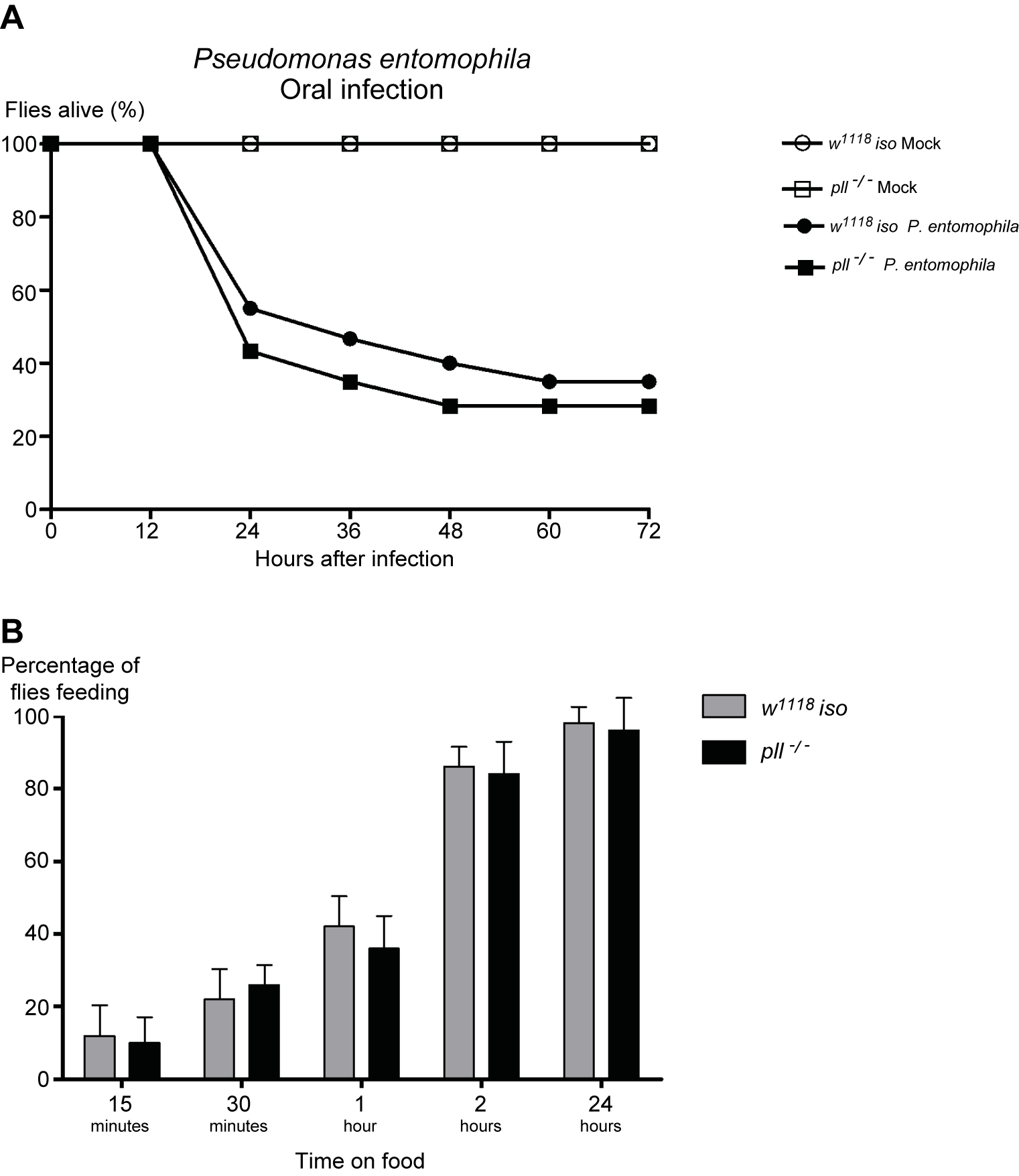

Supplement: Figure S10 — pll mutant and w1118 iso flies have similar sensitivity to Pseudomonas entomophila oral infection and similar ingestion rates. (A) Sixty 3–6 days-old males pll−/− and w1118 iso were orally infected with Pseudomonas entomophila (75 OD) or buffer, and the survival was checked twice a day. Survival data was fitted with a Cox proportional hazard mixed effect model. pll−/− is not significantly different from w1118 iso (p = 0.303). (B) 3–6 days-old pll−/− and w1118 iso males, were exposed to DCV mixed with yeast supplemented with 0,1% bromophenol blue solution. Ingestion rates were measured after 15 min, 30 min, 1 h, 2 h and 24 h by counting flies that had blue abdomens under a dissection microscope. Fifty males per time point were used. Data was fitted with a general linear model. pll−/− mutant and w1118 iso ingestion rates are not different (p = 0.626). (TIF) [file ppat.1004507.s010.tif]

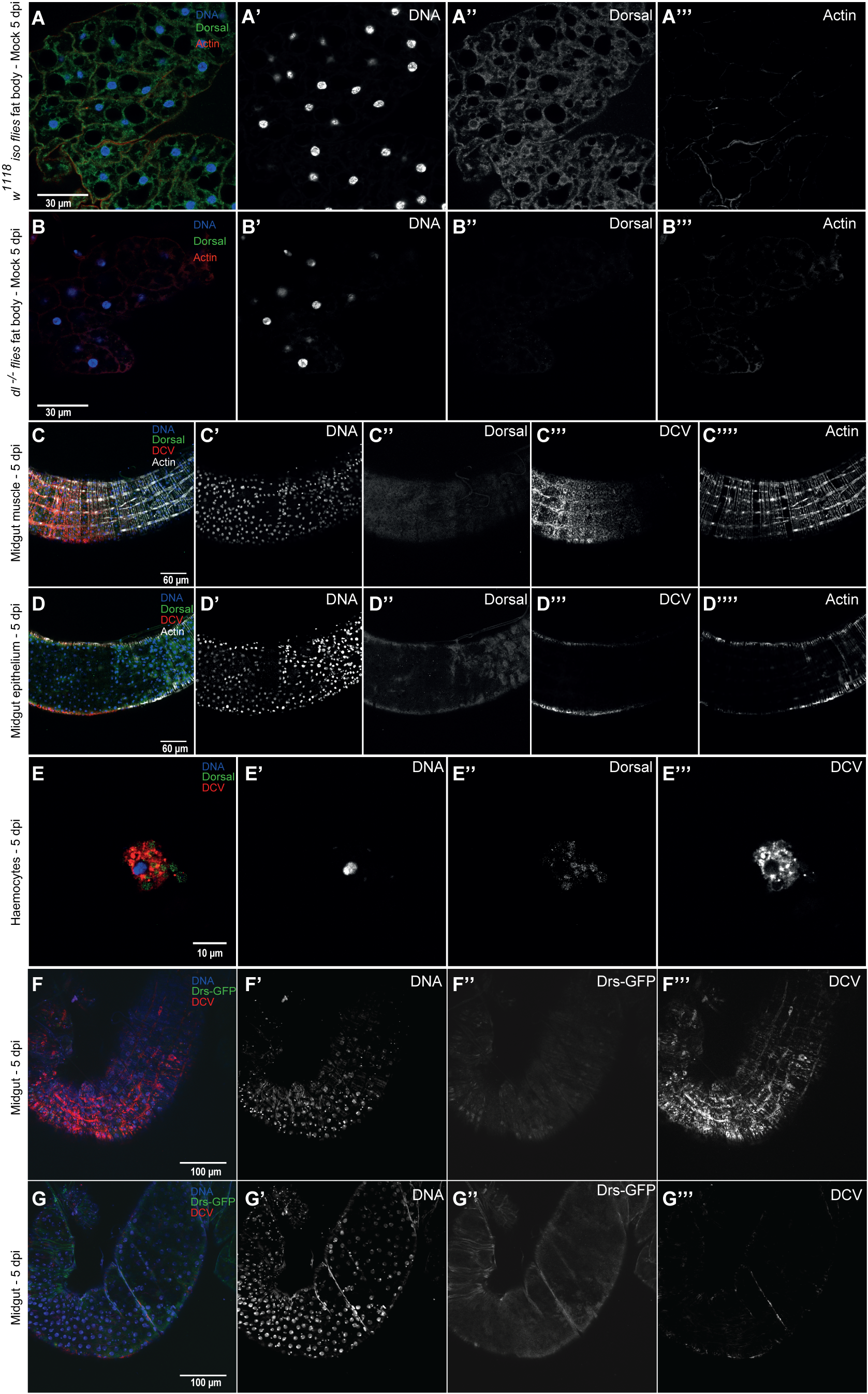

Supplement: Figure S11 — Subcellular localization of Dorsal in fat body and midgut. (A) Lack of Dorsal nuclear import in fat body 5 days after mock oral infection. 6 flies were analysed (B) Absence of Dorsal staining in fat body cells of dl−/− (dl1/dl1) mutant flies, 5 days after mock oral infection. (A–B) Adult male fat body was immunostained with antibody against Dorsal (green), actin marked with phalloidin (red), and DNA marked with DAPI (blue). (C) Midgut muscle cells infected with DCV 5 days after oral infection showing no nuclear import of Dorsal. (D) Midgut epithelial cells 5 days after oral infection showing no nuclear import of Dorsal. (C–D) 14 DCV-positive adult males were analysed, guts were immunostained with an antibody against Dorsal (green), an antibody against DCV (red), actin marked with phalloidin (white) and DNA was marked with DAPI (blue). DCV was at 1011 TCID50/ml. (E) Lack of Dorsal nuclear import in haemocytes 5 days after DCV oral infection. Adult male haemocytes were immunostained with an antibody against Dorsal (green), an antibody against DCV (red) and DNA was marked with DAPI (blue). (F–G) Drs-GFP expression in muscle (F) and epithelium (G) of midgut after 5 days DCV oral infection. (F–G) Adult male midguts were immunostained with antibody against DCV (red), antibody against GFP (green) and DNA marked with TOTO3 (blue). (C–G) DCV dose was 1011 TCID50/ml. (TIF) [file ppat.1004507.s011.tif]

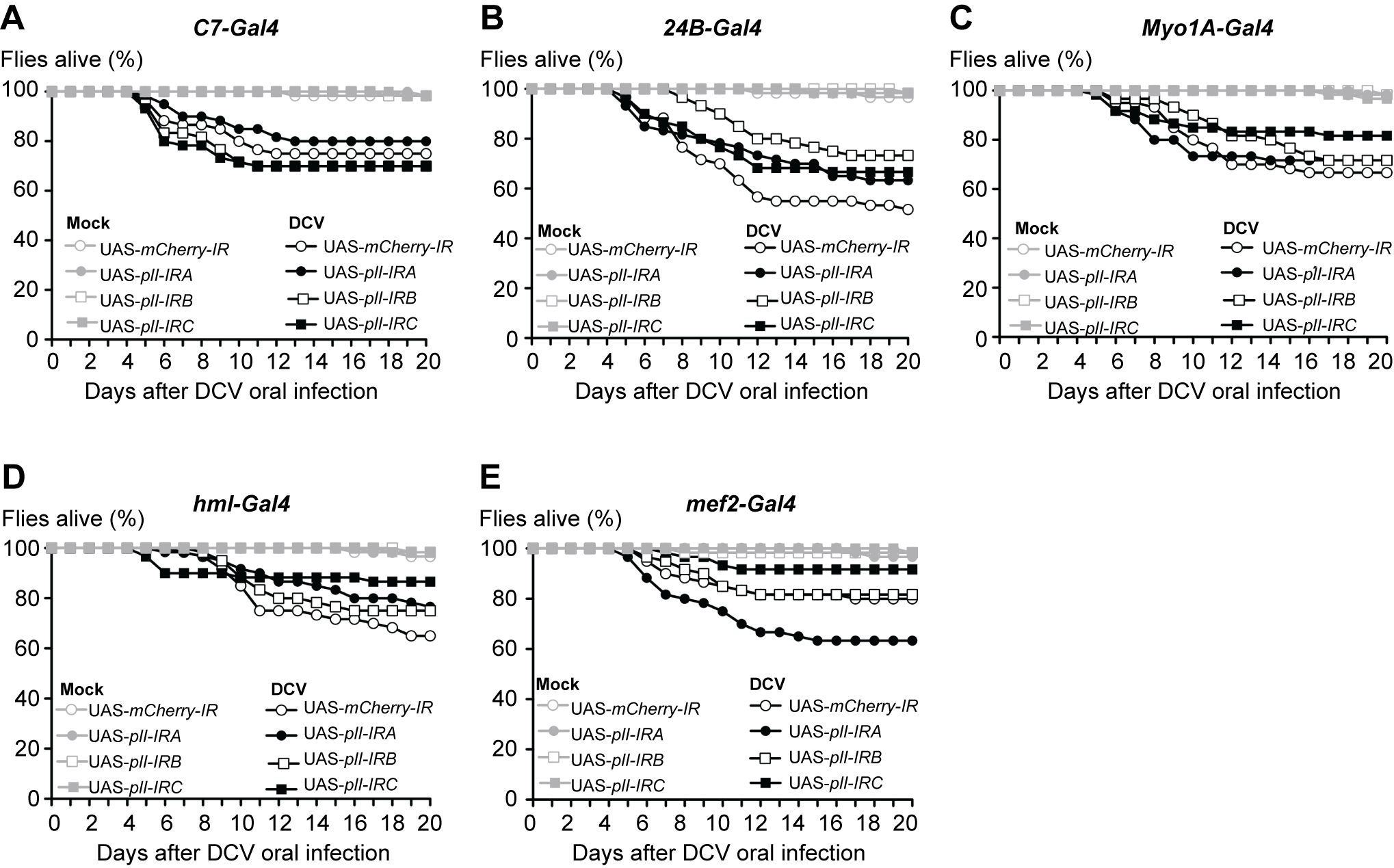

Supplement: Figure S12 — Tissue specific expression of pll RNAi constructs has no effect on survival against oral DCV infection. Survival of three independent UAS-pll-IR constructs and control UAS-mCherry-IR flies upon DCV oral infection (1011 TCID50/ml) or buffer, using tissue specific drivers. Tissue specific UAS-pll-IR expression lines were not more sensitive than control lines, using any of the tested constructs. (A) Fat body specific pll-IR expression using C7-Gal4 (Genotype effect, Cox proportional hazard mixed effect model, p = 0.35). (B) Visceral muscle specific pll-IR expression using 24B-Gal4 driver (Genotype effect, Cox proportional hazard mixed effect model, p = 0.39). (C) Midgut epithelium specific pll-IR expression using Myo1A-Gal4 (Genotype effect, Cox proportional hazard mixed effect model, p = 0.51). (D) Haemocyte specific pll-IR expression using hml(delta)-Gal4 (Genotype effect, Cox proportional hazard mixed effect model, p = 0.12). (E) Somatic, visceral and cardiac muscle specific pll-IR expression using mef2-Gal4 (Genotype effect, Cox proportional hazard mixed effect model, p<0.01; multiple comparisons between UAS-pll-IR lines and UAS-mCherry-IR-line, p>0.43). For all experiments, sixty 3–6 days old males, per line and condition were used, with 10 flies per vial. Flies were orally infected with DCV or buffer, and their survival was monitored daily. Each survival assay was performed twice. Survival data of both replicates was analysed together using Cox proportional hazard mixed effect models. (TIF) [file ppat.1004507.s012.tif]
